# Modeling and Simulation of the Economics of Mining in the Bitcoin Market

**DOI:** 10.1371/journal.pone.0164603

**Published:** 2016-10-21

**Authors:** Luisanna Cocco, Michele Marchesi

**Affiliations:** Department of Electric and Electronic Engineering, University of Cagliari, 09123 Cagliari, Italy; University of Reading, UNITED KINGDOM

## Abstract

In January 3, 2009, Satoshi Nakamoto gave rise to the “Bitcoin Blockchain”, creating the first block of the chain hashing on his computer’s central processing unit (CPU). Since then, the hash calculations to mine Bitcoin have been getting more and more complex, and consequently the mining hardware evolved to adapt to this increasing difficulty. Three generations of mining hardware have followed the CPU’s generation. They are GPU’s, FPGA’s and ASIC’s generations. This work presents an agent-based artificial market model of the Bitcoin mining process and of the Bitcoin transactions. The goal of this work is to model the economy of the mining process, starting from GPU’s generation, the first with economic significance. The model reproduces some “stylized facts” found in real-time price series and some core aspects of the mining business. In particular, the computational experiments performed can reproduce the unit root property, the fat tail phenomenon and the volatility clustering of Bitcoin price series. In addition, under proper assumptions, they can reproduce the generation of Bitcoins, the hashing capability, the power consumption, and the mining hardware and electrical energy expenditures of the Bitcoin network.

## Introduction

Bitcoin is a digital currency alternative to the legal currencies, as any other cryptocurrency. Nowadays, Bitcoin is the most popular cryptocurrency. It was created by a cryptologist known as “Satoshi Nakamoto”, whose real identity is still unknown [1]. Like other cryptocurrencies, Bitcoin uses cryptographic techniques and, thanks to an open source system, anyone is allowed to inspect and even modify the source code of the Bitcoin software.

The Bitcoin network is a peer-to-peer network that monitors and manages both the generation of new Bitcoins and the consistency verification of transactions in Bitcoins. This network is composed by a high number of computers connected to each other through the Internet. They perform complex cryptographic procedures which generate new Bitcoins (mining) and manage the Bitcoin transactions register, verifying their correctness and truthfulness.

Mining is the process which allows to find the so called “proof of work” that validates a set of transactions and adds them to the massive and transparent ledger of every past Bitcoin transaction known as the “Blockchain”. The generation of Bitcoins is the reward for the validation process of the transactions. The Blockchain was generated starting since January 3, 2009 by the inventor of the Bitcoin system himself, Satoshi Nakamoto. The first block is called “Genesis Block” and contains a single transaction, which generates 50 Bitcoins to the benefit of the creator of the block. The whole system is set up to yield just 21 million Bitcoins by 2040, and over time the process of mining will become less and less profitable. The main source of remuneration for the miners in the future will be the fees on transactions, and not the mining process itself.

In this work, we propose an agent-based artificial cryptocurrency market model with the aim to study and analyze the mining process and the Bitcoin market from September 1, 2010, the approximate date when miners started to buy mining hardware to mine Bitcoins, to September 30, 2015.

The model described is built on a previous work of the authors [2], which modeled the Bitcoin market under a purely financial perspective, while in this work, we fully consider also the economics of mining. The proposed model simulates the mining process and the Bitcoin transactions, by implementing a mechanism for the formation of the Bitcoin price, and specific behaviors for each typology of trader who mines, buys, or sells Bitcoins. We calibrated the proposed model by using “blockchain.info”, a web site which displays detailed information about all transactions and Bitcoin blocks, and by tracking the history of the mining hardware. We followed the introduction into the market of the products developed by some mining hardware companies, with the aim to obtain the time trends of the average hash rate per US$ spent on hardware, and of the average power consumption per $\frac{H}{sec}$.

The model was validated studying its ability to reproduce some “stylized facts” found in real-time price series and some core aspects of the real mining business. In particular, the computational experiments performed can reproduce the unit root property, the fat tail phenomenon and the volatility clustering of Bitcoin price series. To our knowledge, this is the first model based on the heterogeneous agents approach that studies the generation of Bitcoins, the hashing capability, the power consumption, and the mining hardware and electrical energy expenditures of the Bitcoin network.

The paper is organized as follows. In Section *Related Work* we discuss other works related to this paper, in Section *Mining Process* we describe briefly the mining process and we give an overview of the mining hardware and of its evolution over time. In Section *The Model* we present the proposed model in detail. Section *Simulation Results* presents the values given to several parameters of the model and reports the results of the simulations, including statistical analysis of Bitcoin real prices and simulated Bitcoin price, and sensitivity analysis of the model to some key parameters. The conclusions of the paper are reported in the last Section. Finally, Appendices A, B, C, and D, in S1 Appendix, deal with the calibration to some parameters of the model, while Appendix E, in S1 Appendix, deals with the sensitivity of the model to some model parameters.

## Related Work

The study and analysis of the cryptocurrency market is a relatively new field. In the latest years, several papers appeared on this topic, given its potential interest and the many issues related to it. Several papers focus on the de-anonymization of Bitcoin users by introducing clustering heuristics to form a user network (see for instance the works [3–5]); others focus on the promise, perils, risks and issues of digital currencies, [6–10]; others focus on the technical issues about protocols and security, [11, 12]. However, very few works were made to model the cryptocurrencies market. Among these, we can cite the works by Luther [13], who studied why some cryptocurrencies failed to gain widespread acceptance using a simple agent model; by Bornholdt and Steppen [14], who proposed a model based on a Moran process to study the cryptocurrencies able to emerge; by Garcia et al. [15], who studied the role of social interactions in the creation of price bubbles; by Kristoufek [16] who analyzed the main drivers of the Bitcoin price; by Kaminsky and Gloor [17] who related the Bitcoin market to its sentiment analysis on social networks; and by Donier and Bouchaud [18] who showed how markets’ crashes are conditioned by market liquidity.

In this paper we propose a complex agent-based artificial cryptocurrency market model in order to reproduce the economy of the mining process, the Bitcoin transactions and the main stylized facts of the Bitcoin price series, following the well known agent-based approach. For reviews about agent-based modelling of the financial markets see the works [19, 20] and [21].

The proposed model simulates the Bitcoin market, studying the impact on the market of three different trader types: Random traders, Chartists and Miners. Random traders trade randomly and are constrained only by their financial resources as in work [22]. They issue buy or sell orders with the same probability and represent people who are in the market for business or investing, but are not speculators. Our Random traders are not equivalent to the so called “noise traders”, who are irrational traders, able of affecting stock prices with their unpredictable changes in their sentiments (see work by Chiarella et al. [23] and by Verma et al. [24]). Chartists represent speculators. They usually issue buy orders when the price is increasing and sell orders when the price is decreasing. Miners are in the Bitcoin market aiming to generate wealth by gaining Bitcoins and are modeled with specific strategies for mining, trading, investing in, and divesting mining hardware. As in the work by Licalzi and Pellizzari [25]—in which the authors model a market where all traders are fundamentalists—the fat tails, one of the main “stylized facts” of the real financial markets, stem from the market microstructure rather than from sophisticated behavioral assumptions.

Note that in our model no trader uses rules to form expectations on prices or on gains, contrarily to the works by Chiarella et al. [23] and by Licalzi and Pellizzari [25], in which traders use rules to form expectations on stock returns. In addition, no trader imitates the expectations of the most successful traders as in the work by Tedeschi et al. [26].

The proposed model implements a mechanism for the formation of the Bitcoin price based on an order book. In particular, the definition of price follows the approach introduced by Raberto et al. [27], in which the limit prices have a random component, modelling the different perceptions of the Bitcoin value, whereas the formation of the price is based on the limit order book, similar to that presented by Raberto et al. [22]. As regards the limit order book, it is constituted by two queues of orders in each instant—sell orders and buy orders. At each simulation step, various new orders are inserted into the respective queues. As soon as a new order enters the book, the first buy order and the first sell order of the lists are inspected to verify if they match. If they match, a transaction occurs. This in contrast with the approach adopted by Chiarella et al. [23], Licalzi and Pellizzari [25] and by Tedeschi et al. [26], in which the agents decide whether to place a buy or a sell order, and choose the size of the order, maximizing their own expected utility function.

The proposed model is, to our knowledge, the first model that aims to study the Bitcoin market and in general a cryptocurrency market– as a whole, including the economics of mining. It was validated by performing several statistical analyses in order to study the stylized facts of Bitcoin price and returns, following the approaches used by Chiarella et al. [23], Cont [28], Licalzi and Pellizzari [25] and Radivojevic et al. [29], for studying the stylized facts of prices and returns in financial markets.

## The Mining Process

Today, every few minutes thousands of people send and receive Bitcoins through the peer-to-peer electronic cash system created by Satoshi Nakamoto. All transactions are public and stored in a distributed database called Blockchain, which is used to confirm transactions and prevent the double-spending problem.

People who confirm transactions of Bitcoins and store them in the Blockchain are called “miners”. As soon as new transactions are notified to the network, miners check their validity and authenticity and collect them into a set of transactions called “block”. Then, they take the information contained in the block, which include a variable number called “nonce”, and run the SHA-256 hashing algorithm on this block, turning the initial information into a sequence of 256 bits, known as Hash [30].

There is no way of knowing how this sequence will look before calculating it, and the introduction of a minor change in the initial data causes a drastic change in the resulting Hash.

The miners cannot change the data containing the information on transactions, but can change the “nonce” number used to create a different hash. The goal is to find a Hash having a given number of leading zero bits. This number can be varied to change the difficulty of the problem. The first miner who creates a proper Hash with success (he finds the “proof-of-work”), gets a reward in Bitcoins, and the successful Hash is stored with the block of the validated transactions in the Blockchain.

In a nutshell,

“Bitcoin miners make money when they find a 32-bit value which, when hashed together with the data from other transactions with a standard hash function gives a hash with a certain number of 60 or more zeros. This is an extremely rare event”, [30].

The steps to run the network are as follows:

“New transactions are broadcast to all nodes; each node collects new transactions into a block; each node works on finding a difficult proof-of-work for its block; when a node finds a proof-of-work, it broadcasts the block to all nodes; nodes accept the block only if all transactions in it are valid and not already spent; nodes express their acceptance of the block by working on creating the next block in the chain, using the hash of the accepted block as the previous hash”, [1].

Producing a single hash is computationally very easy. Consequently, in order to regulate the generation of Bitcoins, the Bitcoin protocol makes this task more and more difficult over time.

The proof-of-work is implemented by incrementing the nonce in the block until a value is found that gives the block’s hash with the required leading zero bits. If the hash does not match the required format, a new nonce is generated and the Hash calculation starts again [1]. Countless attempts may be necessary before finding a nonce able to generate a correct Hash (the size of the nonce is only 32 bits, so in practice it is necessary to vary also other information inside the block to be able to get a hash with the required number of leading zeros, which at the time of writing is about 70).

The computational complexity of the process necessary to find the proof-of-work is adjusted over time in such a way that the number of blocks found each day is more or less constant (approximately 2016 blocks in two weeks, one every 10 minutes). In the beginning, each generated block corresponded to the creation of 50 Bitcoins, this number being halved each four years, after 210,000 blocks additions. So, the miners have a reward equal to 50 Bitcoins if the created blocks belong to the first 210,000 blocks of the Blockchain, 25 Bitcoins if the created blocks range from the 210,001st to the 420,000th block in the Blockchain, 12.5 Bitcoins if the created blocks range from the 420,001st to the 630,000th block in the Blockchain, and so on.

Over time, mining Bitcoin is getting more and more complex, due to the increasing number of miners, and the increasing power of their hardware. We have witnessed the succession of four generations of hardware, i.e. CPU’s, GPU’s, FPGA’s and ASIC’s generation, each of them characterized by a specific hash rate (measured in H/sec) and power consumption. With time, the power and the price of the mining hardware has been steadly increasing, though the price of H/sec has been decreasing. To face the increasing costs, miners are pooling together to share resources.

### The evolution of the mining hardware

In January 3, 2009, Satoshi Nakamoto created the first block of the Blockchain, called “Genesis Block”, hashing on the central processing unit (CPU) of his computer. Like him, the early miners mined Bitcoin running the software on their personal computers. The CPU’s era represents the first phase of the mining process, the other eras being GPU’s, FPGA’s and ASIC’s eras (see web site https://tradeblock.com/blog/the-evolution-of-mining/).

Each era announces the use of a specific typology of mining hardware. In the second era, started about on September 2010, boards based on graphics processing units (GPU) running in parallel entered the market, giving rise to the GPU era.

Around December 2011, the FPGA’s era started, and hardware based on field programmable gate array cards (FPGA) specifically designed to mine Bitcoins was available in the market. Finally, in 2013 fully customized application-specific integrated circuit (ASIC) appeared, substantially increasing the hashing capability of the Bitcoin network and marking the beginning of the fourth era.

Over time, the different mining hardware available was characterized by an increasing hash rate, a decreasing power consumption per hash, and increasing costs. For example, NVIDIA Quadro NVS 3100M, 16 cores, belonging to the GPU generation, has a hash rate equal to 3.6 MH/s and a power consumption equal to 14 W [31]; ModMiner Quad, belonging to the FPGA generation, has a hash rate equal to 800 MH/s and a power consumption equal to 40 W [31]; Monarch(300), belonging to the ASIC generation, has a hash rate equal to 300 GH/s and a power consumption equal to 175 W (see web site https://tradeblock.com/mining/.

### Modelling the Mining Hardware Performances

The goal of our work is to model the economy of the mining process, so we neglected the first era, when Bitcoins had no monetary value, and miners used the power available on their PCs, at almost no cost. We simulated only the remaining three generations of mining hardware.

We gathered information about the products that entered the market in each era to model these three generations of hardware, in particular with the aim to compute:

- the average hash rate per US$ spent on hardware, *R*(*t*), expressed in $\frac{H}{sec*\$}$;
- the average power consumption per *H*/*sec*, *P*(*t*), expressed in $\frac{W}{H/sec}$.

The average hash rate and the average power consumption were computed averaging the real market data at specific times and constructing two fitting curves.

To calculate the hash rate and the power consumption of the mining hardware of the GPU era, that we estimate ranging from September 1st, 2010 to September 29th, 2011, we computed an average for *R* and *P* taking into account some representative products in the market during that period, neglecting the costs of the motherboard.

In that era, motherboards with more than one Peripheral Component Interconnect Express (PCIe) slot started to enter the market, allowing to install multiple video cards in only one system, by using adapters, and to mine criptocurrency, thanks to the power of the GPUs. In Table 1, we describe the features of some GPUs in the market in that period. The data reported are taken from the web site http://coinpolice.com/gpu/.

**Table 1:** GPU Mining Hardware.

Date	Product	Hash Rate GH/$	Consumption W/GH
23/09/2009	Radeon 5830	0.001475	593.22
	Radeon 5850	0.0015	398.94
	Radeon 5870	0.0015	467.66
	Radeon 5970	0.0023	392
22/10/2010	Radeon 6870	0.0015	503.33
	Radeon 6950	0.002	500
	Radeon 6990	0.0018	328.95

As regards the FPGA and ASIC eras, starting around September 2011 and December 2013 respectively, we tracked the history of the mining hardware by following the introduction of Butterfly Labs company’s products into the market. We extracted the data illustrated in Table 2 from the history of the web site http://www.butterflylabs.com/ through the web site web.archive.org. For hardware in the market in 2014 and 2015 we referred to the Bitmain Technologies Ltd company, and in particular, to the mining hardware called AntMiner (see web site https://bitmaintech.com and Table 2).

**Table 2:** Butterfly Labs and Bitmain Technologies Mining Hardware. FPGA Hardware from 09/29/2011 to 12/17/2012, ASIC Hardware from 12/17/2012 to December 2013 and AntMiner Hardware produced in 2014 and 2015.

Date	Product	Price $	Hash Rate GH/s	Hash Rate $\frac{GH}{sec*\$}$	Power Consumption $\frac{W}{GH/sec}$
09/29/2011- 12/2/2011	The Single	699	1	0.0014	19.8
12/2/2011- 12/28/2011	The Single	699	1	0.0014	19.8
	Rig Box	24980	50.4	0.0021	49
12/28/2011- 05/1/2012	The Single	599	0.832	0.0014	96.15
	Rig Box	24980	50.4	0.0021	49
05/1/2012- 12/17/2012	The Single	599	0.832	0.0014	96.15
	Mini Rig	15295	25.2	0.0016	49
12/17/2012- 04/10/2013	BitForce Jalapeno	149	4.5	0.0302	1
	BitForce Little Single SC	649	30	0.0462	1
	BitForce Single SC	1299	60	0.0462	1
	BitForce Mini Rig SC	29899	1500	0.0502	1
04/10/2013- 05/31/2013	Bitcoin Miner	274	5	0.0182	6
	Bitcoin Miner	1249	25	0.02	6
	Bitcoin Miner	2499	50	0.02	6
05/31/2013- 10/15/2013	Bitcoin Miner	274	5	0.0182	6
	Bitcoin Miner	1249	25	0.02	6
	Bitcoin Miner	2499	50	0.02	6
	Bitcoin Miner	22484	500	0.0222	6
10/15/2013- 12/10/2013	Bitcoin Miner	274	5	0.0182	6
	Bitcoin Miner	2499	50	0.02	6
	Bitcoin Miner	22484	500	0.0222	6
	Bitcoin Minin Card	2800	300	0.1071	0.6
	Bitcoin Minin Card	4680	600	0.1282	0.6
12/10/2013- 01/22/2014	AntminerS1	734.18	180	0.245	2
01/22/2014- 07/4/2014	AntminerS2	1715	1000	0.583	1.1
07/4/2014- 10/23/2014	AntminerS4-B2	1250	2000	1.6	0.69
10/23/2014- 03/25/2015	AntminerS5-B5	419	1155	2.756	0.51
03/25/2015-30/09/2015	AntminerS7-B8	454	4730	10.42	0.27

Starting from the mining products in each period (see Tables 1 and 2), we fitted a “best hash rate per $” and a “best power consumption function” (see Table 3). We call the fitting curves *R*(*t*) and *P*(*t*), respectively.

**Table 3:** Average of Hash Rate and of Power Consumption over time.

Date ⇒ Simulation Step	Average of Hash Rate $\frac{GH}{sec*\$}$	Average of power Consumption $\frac{W}{GH/sec}$
September 1, 2010 ⇒ 1	0.0017	454.87
September 29, 2011 ⇒ 394	0.0014	19.8
December 2,2011 ⇒ 458	0.00175	34.4
December 28,2011 ⇒ 484	0.0017	72.575
May 1, 2012 ⇒ 608	0.0029	72.575
December 17, 2012 ⇒ 835	0.03565	1
April 10, 2013 ⇒ 953	0.0194	6
May 31, 2013 ⇒ 1004	0.0201	6
October 15, 2013 ⇒ 1141	0.1351	3.84
December 10, 2013 ⇒ 1197	0.0595	3.84
January 22, 2014 ⇒ 1240	0.245	2
July 4, 2014 ⇒ 1403	0.583	1.1
October 23, 2014 ⇒ 1484	1.6	0.69
March 25, 2015 ⇒ 1667	2.756	0.51
September 30, 2015 ⇒ 1856	10.42	0.27

We used a general exponential model to fit the curve of the hash rate, *R*(*t*) obtained by using Eq (1):

$$\begin{array}{c} R\left(t\right)=a*e^{\left(b*t\right)} \end{array}$$ (1)

where *a* = 8.635*10^4^ and *b* = 0.006318.

The fitting curve of the power consumption *P*(*t*) is also a general exponential model:

$$\begin{array}{c} P\left(t\right)=a*e^{\left(b*t\right)} \end{array}$$ (2)

where *a* = 4.649*10^−7^ and *b* = −0.004055.

**Fig 1:**
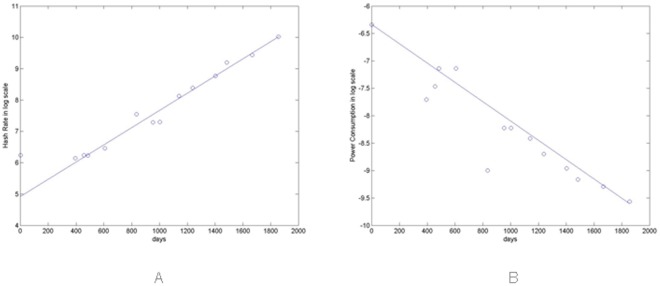
(A) Fitting curve of R(t). (B) fitting curve of P(t).

Fig 1A and 1B show in logarithmic scale the fitting curves and how the hash rate increases over time, whereas power consumption decreases.

## The Model

We used *blockchain.info*, a web site which displays detailed information about all transactions and Bitcoin blocks—providing graphs and statistics on different data—for extracting the empirical data used in this work. In particular, we observed the time trend of the Bitcoin price in the market, the total number of Bitcoins, the total hash rate of the Bitcoin network and the total number of Bitcoin transactions.

The proposed model presents an agent-based artificial cryptocurrency market in which agents mine, buy or sell Bitcoins.

We modeled the Bitcoin market starting from September 1st, 2010, because one of our goals is to study the economy of the mining process. It was only around this date that miners started to buy mining hardware to mine Bitcoins, denoting a business interest in mining. Previously, they typically just used the power available on their personal computers.

The features of the model are:

- there are various kinds of agents active on the BTC market: Miners, Random traders and Chartists;
- the trading mechanism is based on a realistic order book that keeps sorted lists of buy and sell orders, and matches them allowing to fulfill compatible orders and to set the price;
- agents have typically limited financial resources, initially distributed following a power law;
- the number of agents engaged in trading at each moment is a fraction of the total number of agents;
- a number of new traders, endowed only with cash, enter the market; they represent people who decided to start trading or mining Bitcoins;
- Miners belong to mining pools. This means that at each time *t* they always have a positive probability to mine at least a fraction of Bitcoin. Indeed, since 2010 miners have been pooling together to share resources in order to avoid effort duplication to optimally mine Bitcoins. A consequence of this fact is that gains are smoothly distributed amongst Miners.
  On July 18th, 2010,

“ArtForz establishes an OpenGL GPU hash farm and generates his first Bitcoin block”

and on September 18th, 2010,

“Bitcoin Pooled Mining (operated by slush), a method by which several users work collectively to mine Bitcoins and share in the benefits, mines its first block”,

(news from the web site http://historyofBitcoin.org/).

Since then, the difficulty of the problem of mining increased exponentially, and nowadays it would be almost unthinkable to mine without participating in a pool.

In the next subsections we describe the model simulating the mining, the Bitcoin market and the related mechanism of Bitcoin price formation in detail.

### The Agents

Agents, or traders, are divided into three populations: Miners, Random traders and Chartists.

Every *i*-th trader enters the market at a given time step, $t_{i}^{E}$. Such a trader can be either a Miner, a Random trader or a Chartist. All traders present in the market at the initial time $t_{i}^{E}=0$ hold an amount *c*_*i*_(0) of fiat currency (cash, in US dollars) and an amount *b*_*i*_(0) of cryptocurrency (Bitcoins), where *i* is the trader’s index. They represent the persons present in the market, mining and trading Bitcoins, before the period considered in the simulation. Each *i*-th trader entering the market at $t_{i}^{E}>0$ holds only an amount $c_{i}\left(t_{i}^{E}\right)$ of fiat currency (cash, in dollars). These traders represent people interested in entering the market, investing their money in it.

The wealth distribution of traders follows a Zipf law [32]. The set of all traders entering the market at time $t_{i}^{E}>0$ are generated before the beginning of the simulation with a Pareto distribution of fiat cash, and then are randomly extracted from the set, when a given number of them must enter the market at a given time step. Also, the wealth distribution in crypto cash of the traders in the market at initial time follows a Zipf law. Indeed, the wealth share in the world of Bitcoin is even more unevenly distributed than in the world at large (see web site http://www.cryptocoinsnews.com/owns-Bitcoins-infographic-wealth-distribution/). More details on the trader wealth endowment are illustrated in *Appendix A*, in S1 Appendix. In that appendix, we report also some results that show that the heterogeneity in the fiat and crypto cash of the traders emerges endogenously also when traders start from the same initial wealth.

#### Miners

*Miners* are in the Bitcoin market aiming to generate wealth by gaining Bitcoins. At the initial time, the simulated Bitcoin network is calibrated according to Satoshi’s original idea of Bitcoin network, where each node participates equally to the process of check and validation of the transactions and mining. We assumed that Miners in the market at initial time ($t_{i}^{E}=0$) own a Core i5 2600K PC, and hence they are initially endowed with a hashing capability *r*_*i*_(0) equal to 0.0173GH/sec, that implies a power consumption equal to 75W [31]. Core i5 is a brand name of a series of fourth-generation x64 microprocessors developed by Intel and brought to market in October 2009.

Miners entering the market at time $t_{i}^{E}>0$ acquire mining hardware, and hence a hashing capability *r*_*i*_(*t*)—which implies a specific electricity cost *e*_*i*_(*t*)—investing a fraction *γ*_1,*i*_(*t*) of their fiat cash *c*_*i*_(*t*).

In addition, over time all Miners can improve their hashing capability by buying new mining hardware investing both their fiat and crypto cash. Consequently, the total hashing capability of *i*–*th* trader at time *t*, *r*_*i*_(*t*) expressed in [*H*/*sec*], and the total electricity cost *e*_*i*_(*t*) expressed in $ per day, associated to her mining hardware units, are defined respectively as:

$$\begin{array}{c} r_{i}\left(t\right)=\sum_{s=t_{i}^{E}}^{t}r_{i,u}\left(t\right) \end{array}$$ (3)

and

$$\begin{array}{c} e_{i}\left(t\right)=\sum_{s=t_{i}^{E}}^{t}\epsilon *P\left(s\right)*r_{i,u}\left(s\right)*24 \end{array}$$ (4)

where:

$$\begin{array}{c} r_{i,u}\left(t=t_{i}^{E}>0\right)=\gamma_{1,i}\left(t\right)c_{i}\left(t\right)R\left(t\right) \end{array}$$ (5)

$$\begin{array}{c} r_{i,u}\left(t>t_{i}^{E}\right)=\left[\gamma_{1,i}\left(t\right)c_{i}\left(t\right)+\gamma_{i}\left(t\right)b_{i}\left(t\right)p\left(t\right)\right]R\left(t\right) \end{array}$$ (6)

- *R*(*t*) and *P*(*t*) are, respectively, the hash rate which can be bought with one US$, expressed in $\frac{H}{sec*\$}$, and the power consumption, expressed in $\frac{W}{H/sec}$. At each time *t*, their values are given by using the fitting curves described in subsection *Modelling the Mining Hardware Performances*;
- *r*_*i*,*u*_(*t*) is the hashing capability of the hardware units *u* bought at time *t* by *i*–*th* miner;
- *γ*_*i*_(*t*) = 0 and *γ*_1,*i*_(*t*) = 0 if no hardware is bought by *i*–*th* trader at time *t*. When a trader decides to buy new hardware, *γ*_1,*i*_ represents the percentage of the miner’s cash allocated to buy it. It is equal to a random variable characterized by a lognormal distribution with average 0.6 and standard deviation 0.15. *γ*_*i*_ represents the percentage of the miner’s Bitcoins to be sold for buying the new hardware at time *t*. It is equal to 0.5*γ_1,*i*_(*t*). The term *γ*_1,*i*_(*t*)*c*_*i*_(*t*) + *γ*_*i*_(*t*)*b*_*i*_(*t*)*p*(*t*) expresses the amount of personal wealth that the miner wishes to allocate to buy new mining hardware, meaning that on average the miner will allocate 60% of her cash and 30% of her Bitcoins to this purpose. If *γ*_*i*_ > 1 or *γ*_1,*i*_ > 1, they are set equal to one;
- *ϵ* is the fiat price per Watt and per hour. It is assumed equal to 1.4*10^−4^ $, considering the cost of 1 KWh equal to 0.14$, which we assumed to be constant throughout the simulation. This electricity price is computed by making an average of the electricity prices in the countries in which the Bitcoin nodes distribution is higher; see web sites https://getaddr.bitnodes.io and http://en.wikipedia.org/wiki/Electricity_pricing.

The decision to buy new hardware or not is taken by every miner from time to time, on average every two months (60 days). If *i*–*th* miner decides whether to buy new hardware and/or to divest the old hardware units at time *t*, the next time, $t_{i}^{I-D}\left(t\right)$, she will decide again is given by Eq (7):

$$\begin{array}{c} t_{i}^{I-D}\left(t\right)=t+int\left(60+N\left(\mu^{id},\sigma^{id}\right)\right) \end{array}$$ (7)

where *int* rounds to the nearest integer and *N*(*μ*^*id*^,*σ*^*id*^) is a normal distribution with average *μ*^*id*^ = 0 and standard deviation *σ*^*id*^ = 6. $t_{i}^{I-D}\left(t\right)$ is updated each time the miner takes her decision.

Miners active in the simulation since the beginning will take their first decision within 60 days, at random times uniformly distributed. Miners entering the simulation at time *t* > 1 will immediately take this decision.

In deeper detail, at time $t=t_{i}^{I-D}\left(t\right)$, every miner buys new hardware units, if their fiat cash is positive, and divests the hardware units older than one year. This is because, in general, Bitcoin mining hardware become obsolete from a few months to one year after you purchase it. “Serious” miners usually buy new equipment every month, re-investing their profits into new mining equipment, if they want their Bitcoin mining operation to run long term (see web site http://coinbrief.net/profitable-bitcoin-mining-farm/. If the trader’s cash is zero, she issues a sell market order to get the cash to support her electricity expenses, *c*_*i*,*a*_(*t*) = *γ*_*i*_(*t*)*b*_*i*_(*t*)*p*(*t*).

Each *i*–*th* miner belongs to a pool, and consequently at each time *t* she always has a probability higher than 0 to mine at least some sub-units of Bitcoin. This probability is inversely proportional to the hashing capability of the whole network. Knowing the number of blocks discovered per day, and consequently knowing the number of new Bitcoins *B* to be mined per day, the number of Bitcoins *b*_*i*_ mined by *i*–*th* miner per day can be defined as follows:

$$\begin{array}{c} b_{i}\left(t\right)=\frac{r_{i}\left(t\right)}{r_{Tot}\left(t\right)}B\left(t\right) \end{array}$$ (8)

where:

- *r*_*Tot*_(*t*) is the hashing capability of the whole population of Miners *N*_*m*_ at time *t* defined as the sum of the hashing capabilities of all Miners at time *t*, $r_{Tot}\left(t\right)=\sum_{i}^{N_{m}}r_{i}\left(t\right)$;
- the ratio $\frac{r_{i}\left(t\right)}{r_{Tot}\left(t\right)}$ defines the relative hash rate of *i*–*th* miner at time *t*.

Note that, as already described in the section *Mining Process*, the parameter *B* decreases over time. At first, each generated block corresponds to the creation of 50 Bitcoins, but after four years, such number is halved. So, until November 27, 2012, 100,800 Bitcoins were mined in 14 days (7200 Bitcoins per day), and then 50,400 Bitcoins in 14 days (3600 per day).

#### Random Traders

*Random traders* represent persons who enter the cryptocurrency market for various reasons, but not for speculative purposes. They issue orders for reasons linked to their needs, for instance they invest in Bitcoins to diversify their portfolio, or they disinvest to satisfy a need for cash. They issue orders in a random way, compatibly with their available resources. In particular, buy and sell orders are always issued with the same probability. The specifics of their behavior are described in section *Buy and Sell Orders*.

#### Chartists

*Chartists* represent speculators, aimed to gain by placing orders in the Bitcoin market. They speculate that, if prices are rising, they will keep rising, and if prices are falling, they will keep falling. In particular, *i*–*th* Chartist issues a buy order when the price relative variation in a time window $\tau_{i}^{C}$, is higher than a threshold *Th*^*C*^ = 0.01, and issues a sell order if this variation is lower than *Th*^*C*^. $\tau_{i}^{C}$ is specific for each Chartist, and is characterized by a normal distribution with average equal to 20 and standard deviation equal to 1. Chartists usually issue buy orders when the price is increasing and sell orders when the price is decreasing.

Note that a Chartist will issue an order only when the price variation is above a given threshold. So, in practice, the extent of Chartist activity varies over time.

All Random traders and Chartists entering the market at *t* = *t*^*E*^ > 0, issue a buy order to acquire their initial Bitcoins. Over time, at time *t* > *t*^*E*^ only a fraction of Random traders and Chartists is active, and hence enabled to issue orders. Active traders can issue only one order per time step, which can be a sell order or a buy order.

Orders already placed but not yet satisfied or withdrawn are accounted for when determining the amount of Bitcoins a trader can buy or sell. Details on the percentage of active traders, the number of the traders in the market and on the probability of each trader to belong to a specific traders’ population are described in Appendices B, C, and D, in S1 Appendix.

### Buy and Sell Orders

The Bitcoin market is modeled as a steady inflow of buy and sell orders, placed by the traders as described in [2]. Both buy and sell orders are expressed in Bitcoins, that is, they refer to a given amount of Bitcoins to buy or sell. In deeper detail, all orders have the following features:

- amount, expressed in $ for buy order and in Bitcoins for sell order: the latter amount is a real number, because Bitcoins can be bought and sold in fractions as small as a “Satoshi”;
- residual amount (Bitcoins or $): used when an order is only partially satisfied by previous transactions;
- limit price (see below), which in turn can be a real number;
- time when the order was issued;
- expiration time: if the order is not (fully) satisfied, it is removed from the book at this time.

The amount of each buy order depends on the amount of cash, *c*_*i*_(*t*), owned by *i*-th trader at time *t*, less the cash already committed to other pending buy orders still in the book. Let us call $c_{i}^{b}$ the available cash. The number of Bitcoins to buy, *b*_*a*_ is given by Eq (9)

$$\begin{array}{c} b_{a}=\frac{c_{i}^{b}\beta }{p(t)} \end{array}$$ (9)

where *p*(*t*) is the current price and *β* is a random variable drawn from a lognormal distribution with average and standard deviation equal to 0.25 and 0.2, respectively for Random traders and equal to 0.4 and 0.2, respectively for Chartists. In the unlikely case that *β* > 1, *β* is set equal to 1.

Similarly, the amount of each sell order depends on the number of Bitcoins, *b*_*i*_(*t*) owned by *i*-th trader at time *t*, less the Bitcoins already committed to other pending sell orders still in the book, overall called $b_{i}^{s}$. The number of Bitcoins to sell, *s*_*a*_ is given by

$$\begin{array}{c} s_{a}=b_{i}^{s}\beta \end{array}$$ (10)

where *β* is a lognormal random variable as above. Short selling is not allowed.

The limit price models the price to which a trader desires to conclude their transaction. An order can also be issued with no limit (market order), meaning that its originator wishes to perform the trade at the best price she can find. In this case, the limit price is set to zero. The probability of placing a market order, *P*_*lim*_, is set at the beginning of the simulation and is equal to 1 for Miners, to 0.2 for Random traders and to 0.7 for Chartists. This is because, unlike Random traders, if Miners and Chartists issue orders, they wish to perform the trade at the best available price, the former because they need cash, the latter to be able to profit by following the price trend.

Let us suppose that *i*-th trader issues a limit order to buy $a_{i}^{b}\left(t\right)$ Bitcoins at time *t*. Each buy order can be executed if the trading price is lower than, or equal to, its buy limit price *b*_*i*_. In the case of a sell order of $a_{i}^{s}\left(t\right)$ Bitcoins, it can be executed if the trading price is higher than, or equal to, its sell limit price *s*_*i*_. As said above, if the limit prices *b*_*i*_ = 0 or *s*_*i*_ = 0, then the orders can be always executed, provided there is a pending complementary order.

The buy and sell limit prices, *b*_*i*_ and *s*_*i*_, are given respectively by the following equations:

$$\begin{array}{c} b_{i}\left(t\right)=p\left(t\right)*N_{i}\left(\mu ,\sigma_{i}\right) \end{array}$$ (11)

$$\begin{array}{c} s_{i}\left(t\right)=\frac{p(t)}{N_{i}\left(\mu ,\sigma_{i}\right)} \end{array}$$ (12)

where

- *p*(*t*) is the current Bitcoin price;
- $N_{i}\left(\mu ,\sigma_{i}^{c}\right)$ is a random draw from a Gaussian distribution with average *μ* ≃ 1 and standard deviation *σ*_*i*_ ≪ 1.

The limit prices have a random component, modelling the different perception of Bitcoin value, that is the fact that what traders “feel” is the right price to buy or to sell is not constant, and may vary for each single order. In the case of buy orders, we stipulate that a trader wishing to buy must offer a price that is, on average, slightly higher than the market price.

The value of *σ*_*i*_ is proportional to the “volatility” *σ*(*T*_*i*_) of the price *p*(*t*) through the equation *σ*_*i*_ = *Kσ*(*T*_*i*_), where *K* is a constant and *σ*(*T*_*i*_) is the standard deviation of price absolute returns, calculated in the time window *T*_*i*_. *σ*_*i*_ is constrained between a minimum value *σ*_*min*_ and a maximum value *σ*_*max*_ (this is an approach similar to that of [27]). For buy orders *μ* = 1.05, *K* = 2.5, *σ*_*min*_ = 0.01 and *σ*_*max*_ = 0.003.

In the case of sell orders, the reasoning is dual. For symmetry, the limit price is divided by a random draw from the same Gaussian distribution $N_{i}\left(\mu ,\sigma_{i}^{c}\right)$.

An expiration time is associated to each order. For Random traders, the value of the expiration time is equal to the current time plus a number of days (time steps) drawn from a lognormal distribution with average and standard deviation equal to 3 and 1 days, respectively. In this way, most orders will expire within 4 days since they were posted. Chartists, who act in a more dynamic way to follow the market trend, post orders whose expiration time is at the end of the same trading day. Miners issue market orders, so the value of the expiration time is set to infinite.

### Price Clearing Mechanism

We implemented the price clearing mechanism by using an Order Book similar to that presented in [22].

At every time step, the order book holds the list of all the orders received and still to be executed. Buy orders are sorted in descending order with respect to the limit price *b*_*i*_. Sell orders are sorted in ascending order with respect to the limit price *s*_*j*_. Orders with the same limit price are sorted in ascending order with respect to the order issue time.

At each simulation step, various new orders are inserted into the respective lists. As soon as a new order enters the book, the first buy order and the first sell order of the lists are inspected to verify if they match. If they match, a transaction occurs. The order with the smallest residual amount is fully executed, whereas the order with the largest amount is only partially executed, and remains at the head of the list, with its residual amount reduced by the amount of the matching order. Clearly, if both orders have the same residual amount, they are both fully executed.

After the transaction, the next pair of orders at the head of the lists are checked for matching. If they match, they are executed, and so on until they do not match anymore. Hence, before the book can accept new orders, all the matching orders are satisfied.

A sell order of index *j* matches a buy order of index *i*, and vice versa, only if *s*_*j*_ ≤ *b*_*i*_, or if one of the two limit prices, or both, are equal to zero.

As regards the price, *p*_*T*_, to which the transaction is performed, the price formation mechanism follows the rules described below. Here, *p*(*t*) denotes the current price:

- when one of the two orders has limit price equal to zero:
  - if *b*_*i*_ > 0, then *p*_*T*_ = *min*(*b*_*i*_,*p*(*t*)),
  - if *s*_*j*_ > 0, then *p*_*T*_ = *max*(*s*_*j*_,*p*(*t*)),
- when both orders have limit price equal to zero, *p*_*T*_ = *p*(*t*);
- when both orders have limit price higher than zero, $p_{T}=\frac{b_{i}+s_{j}}{2}$.

## Simulation Results

The model described in the previous section was implemented in Smalltalk language. Before the simulation, it had to be calibrated in order to reproduce the real stylized facts and the mining process in the Bitcoin market in the period between September 1st, 2010 and September 30th, 2015. The simulation period was thus set to 1856 steps, a simulation step corresponding to one day. We included also weekends and holidays, because the Bitcoin market is, by its very nature, accessible and working every day.

Some parameter values are taken from the literature, others from empirical data, and others are guessed using common sense, and tested by verifying that the simulation outputs were plausible and consistent. We set the initial value of several key parameters of the model by using data recovered from the Blockchain Web site. The main assumption we made is to size the artificial market at about 1/100 of the real market, to be able to manage the computational load of the simulation. Table 4 shows the values of some parameters and their computation assumptions in detail. Other parameter values are described in the description of the model presented in the Section *The Model*. In Appendices A-D, in S1 Appendix, other details about the calibration of the model are shown. Specifically, the calibration of the trader wealth endowment, the number of active traders, the total number of traders in the market and the probability of a trader to belong to a specific traders’ population are described in detail.

**Table 4:** Values of some simulation parameters and the assumptions behind them.

Param.	Initial Value	Description and discussion
*N*_*t*_(0)	160	Number of initial traders. Obtained dividing the number of traders on September 1st, 2010 estimated through the fitting curve shown in Eq (1) by 100 (see Appendix B in S1 Appendix).
*N*_*t*_(*T*)	39,649	Total number of traders at the end of the simulation. Obtained dividing the number of traders on September 30, 2015 estimated through the fitting curve shown in Eq (1) by 100 (see Appendix B in S1 Appendix).
*B*	72 or 36	Bitcoins mined per day. Obtained dividing the Bitcoins mined every day by 100. They are 72 until 853*th* simulation step (November 27th, 2012), and 36 from 853*th* simulation step onwards.
*p*(0)	0.0649 $	Initial price. The average price as of September 2010.
*B*_*T*_(0)	23,274 $	Total initial crypto cash. Obtained dividing the number of Bitcoins on September 1st, 2010 by 100 and keeping just 60% of this value, because we assume that 40% of Bitcoins are not available for trade.
*q*	200,000 $	Constant used in Zipf’s law ($\frac{q}{i^{0.6}}$), used to assign the initial cash for traders entering at *t* > 1.
$c_{1}^{s}$	20,587 $	Initial cash of the richest trader entering the simulation at *t* = 1.
$b_{1}^{s}$	4,117 $	Initial Bitcoin amount of the richest trader entering the simulation at *t* = 1.

The model was run to study the main features of the Bitcoin market and of the traders who operate in it. In order to assess the robustness of our model and the validity of our statistical analysis, we repeated 100 simulations with the same initial conditions, but different seeds of the random number generator. The results of all simulations were consistent, as the following shows.

### Bitcoin prices in the real and simulated market

**Fig 2:**
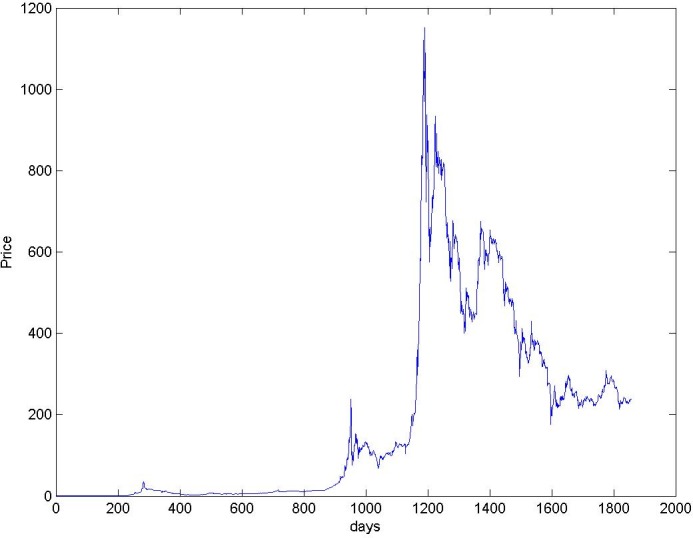
Price of Bitcoins in US$.

We started studying the real Bitcoin price series between September 1st, 2010 and September 30, 2015, shown in Fig 2. The figure shows an initial period in which the price trend is relatively constant, until about 950^*th*^ day. Then, a period of volatility follows between 950^*th*^ and 1150^*th*^ day, followed by a period of strong volatility, until the end of the considered interval. The Bitcoin price started to fall at the beginning of 2014, and continued on its downward slope until September 2015.

**Fig 3:**
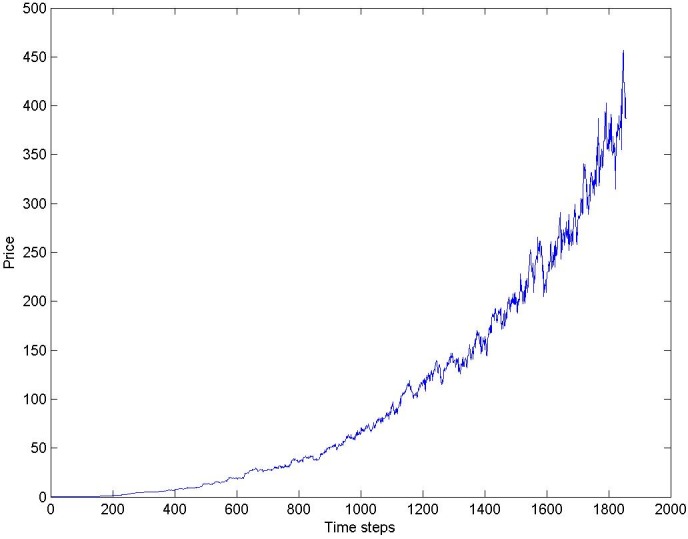
Bitcoin simulated Price in one simulation run.

As regards the prices in the simulated market, we report in Fig 3 the Bitcoin price in one typical simulation run. It is possible to observe that, as in the case of the real price, the price keeps its value constant at first, but then, after about 1000 simulation steps, contrary to what happens in reality, it grows and continues on its upward slope until the end of the simulation period.

**Fig 4:**
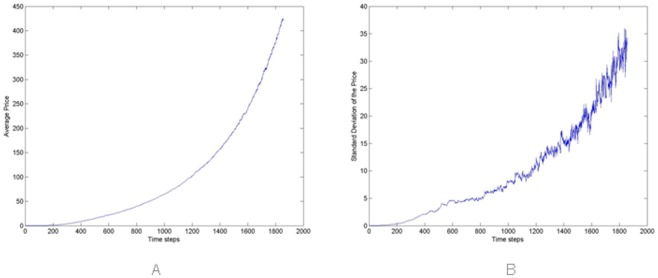
(A) Average Price and (B) standard deviation computed on the 100 Monte Carlo simulations performed.

Fig 4A and 4B report the average and the standard deviation of the price in the simulated market, taken on all 100 simulations. Note that the average value of prices steadily increases with time, except for short periods, in contrast with what happens in reality. Fig 4B shows that the price variations in different simulation runs increase with time, as the number of traders, transactions and the total wealth in the market are increasing.

In the proposed model, the upward trend of the price depends on an intrinsic mechanism—the average price tends to the ratio of total available cash to total available Bitcoins. Since new traders bring in more cash than newly mined Bitcoins, the price tends to increase.

In reality, Bitcoin price is also heavily affected by exogenous factors. For instance, in the past the price strongly reacted to reports such as those regarding the Bitcoin ban in China, or the MtGox exchange going bust. Moreover, the total capitalization of the Bitcoin market is of the order of just some billion US$, so if a large hedge fund decided to invest in Bitcoins, or if large amounts of Bitcoins disappeared because of theft, fraud or mismanagement, the effect on price would potentially be very large. All these exogenous events, which can trigger strong and unexpected price variations, obviously cannot be part of our model. However, the validity of these agent-based market models is typically validated by their ability to reproduce the statistical properties of the price series, which is the subject of the next section.

### Statistical analysis of Bitcoin prices in the real and simulated markets

Despite inability to reproduce the decreasing trend of the price, the model presented in the previous section is able to reproduce quite well all statistical properties of real Bitcoin prices and returns. The stylized facts, robustly replicated by the proposed model, are the same of a previous work of Cocco et al. [2].

It is well known that the price series encountered in financial markets typically exhibit some statistical features, also known as “stylized facts” [33, 34]. Among these, the three uni-variate properties that appear to be the most important and pervasive of price series, are (i) the unit-root property, (ii) the fat tail phenomenon, and (iii) the *Volatility Clustering*. We examined daily Bitcoin prices in real and simulated markets, and found that also these prices exhibit these properties as discussed in detail in [2].

Regarding unit-root property, it amounts to being unable to reject the hypothesis that financial prices follow a random walk. To this purpose, we applied the Augmented Dickey-Fuller test, under the null hypothesis of random walk without drift, to the series of Bitcoin daily prices and to the series of Bitcoin daily price logarithms we considered. The corresponding critical values of the *τ*_1_ statistic for the null hypothesis of random walk without drift at levels 1, 5, and 10% with 1856 observations are −2.58, −1.95 and −1.62 respectively. The *τ*_1_ statistic is -1.2, and 0.5, respectively, for price series and price logarithm series. Consequently, at levels 1, 5, and 10% we cannot reject the null hypothesis.

The second property is the fat-tail phenomenon. Typically, in financial markets the distribution of returns at weekly, daily and higher frequencies displays a heavy tail with positive excess kurtosis.

The Kurtosis value of the real price returns is equal to 125.1 (see Table 5), consequently the distribution of returns is more outlier-prone than the normal distribution. The distribution of returns is a leptokurtic distribution, and so we can infer a “fat tail”.

**Table 5:** Descriptive statistics of the real price returns and of the real price absolute returns in brackets.

Descriptive statistics	Value
mean	0.007 (0.04)
st. dev	0.08 (0.07)
skewness	6.9 (10)
kurtosis	125.1 (176)

**Fig 5:**
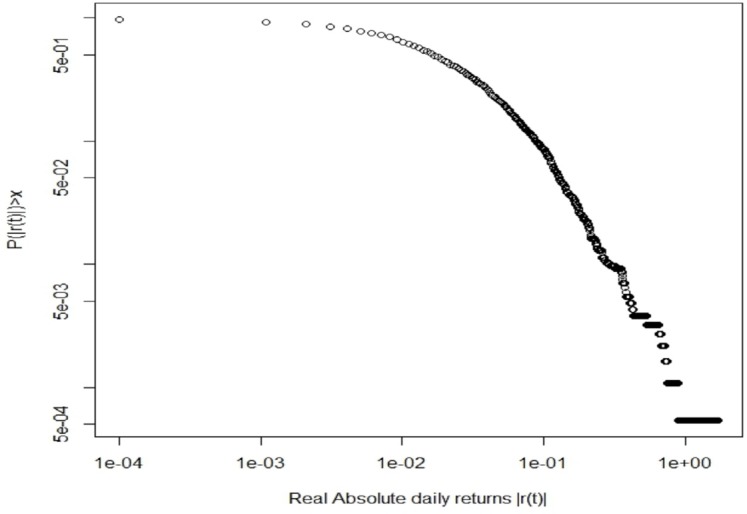
The decumulative distribution function of the absolute returns.

Fig 5 shows the decumulative distribution function of the absolute returns (DDF), that is the probability of having a chance in price larger than a given return threshold. This is the plot of one minus the cumulative distribution function of the absolute returns and highlights a “fat tail”.

To confirm the above statements, we also computed the Hill tail index. This is a measure of the power-law tail exponent, *α*; the lower *α*, the fatter the tail of the DDF. Hill index is computed through Eq (13) [35][36]:

$$\begin{array}{c} \alpha =1+n{\left\{\sum_{i=1}^{n}ln\left(\frac{x_{i}}{x_{min}}\right)\right\}}^{-1} \end{array}$$ (13)

where *x*_*i*_ is the daily return and *x*_*min*_ corresponds to the smallest value of *x*_*i*_ for which the power-law behavior holds, set equal to 0.05 in our analysis.

The index takes a value equal to 2.48, and is in accordance with those of real financial markets, where this index is normally below 4, as stated by Lux [37]. We also found that the right tail (due to positive changes in returns) of the distribution is fatter than the left tail (due to negative changes in returns). These indexes take values equal to 2.34 and 2.75, respectively. This is in contradiction with the situation in real financial markets, where the tail due to negative returns is fatter than the one due to positive returns [37].

The third property is *Volatility Clustering*: periods of quiescence and turbulence tend to cluster together. This can be verified by the presence of highly significant autocorrelation in absolute or squared returns, despite insignificant autocorrelation in raw returns.

**Fig 6:**
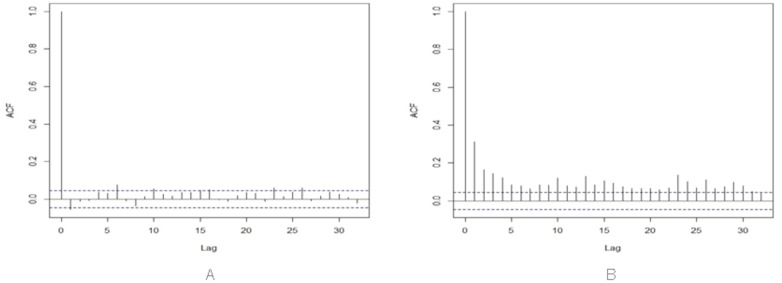
Autocorrelation of (A) raw returns, and (B) absolute returns of Bitcoin prices.

Fig 6B and 6C show the autocorrelation functions of the real price returns and absolute returns, at time lags between zero and 20. It is possible to note that the autocorrelation of raw returns Fig 6B is often negative, and is anyway very close to zero, whereas the autocorrelation of absolute returns Fig 6C has values significantly higher than zero. This behavior is typical of financial price return series, and confirms the presence of volatility clustering.

In conclusion, the Bitcoin price shows all the stylized facts of financial price series, as expected.

As regards the simulated market model, all statistical properties of real prices and returns are reproduced quite well in our model.

In Table 6 the percentiles of the *τ*_1_ statistic for the null hypothesis of random walk without drift across all Monte Carlo simulations, varying of the parameter *Th*^*C*^, are described. Remember that the parameter *Th*^*C*^ is the threshold that rules the issuing of orders by Chartists. The *i*–*th* Chartist issues a buy order when the price relative variation in a time window $\tau_{i}^{C}$, is higher than a threshold *Th*^*C*^ = 0.01, and issues a sell order if this variation is lower than *Th*^*C*^. Note that for *Th*^*C*^ = ∞ no Chartist is active in the market. For each value of the parameter *Th*^*C*^, and at each level they are always higher than the corresponding critical value, so also for the simulated data we cannot reject the null hypothesis of random walk of prices.

**Table 6:** Percentile Values of the *τ*_1_ statistic for the null hypothesis of random walk without drift across all Monte Carlo simulations, varying of the parameter *Th*^*C*^. Statistics of price logarithm series are in brackets.

	Percentile Value				
*Th*^*C*^	0.01	.25	.50	.75	.975
0.01	1.18 (2.34)	1.81 (2.61)	2.24 (2.77)	2.65 (2.88)	3.22 (3.2)
0.02	1.12 (2.16)	1.8 (2.63)	2.4 (2.77)	2.6 (2.84)	3.2 (2.96)
0.05	1.88 (2.52)	2.01 (2.71)	2.08 (2.8)	2.13 (2.85)	2.6 (2.9)
∞	1.18 (2.04)	1.58 (2.18)	1.81 (2.34)	2.05 (2.51)	2.53 (2.75)

In Table 7, the 25th, 50th, 75th and 97.5th percentiles pertaining to average, standard deviation, skewness and kurtosis of the price returns across all Monte Carlo simulations are shown. The values of the mean of price returns and of absolute returns, as well as their standard deviations, compare well with the real values. The skewness of simulated prices tends to be lower than the real case but it is always positive. The simulated kurtosis is lower than the real case by more than one order of magnitude, but also for the simulated price returns we can infer a fat tail for their distribution.

**Table 7:** Percentile Values of some descriptive statistics of the price returns and of the price absolute returns in brackets across all Monte Carlo simulations.

Descriptive statistics	Percentile Value			
	.25	.50	.75	.975
mean	0.0052 (0.0228)	0.0053 (0.023)	0.0053 (0.024)	0.0054 (0.025)
st. dev	0.032 (0.023)	0.033 (0.024)	0.034 (0.025)	0.036 (0.027)
skewness	0.4 (2.05)	0.6 (2.4)	0.81 (2.6)	2 (5)
kurtosis	6 (9.9)	7.6 (12)	9 (17)	25 (58)

We computed the Hill tail index, and also the Hill index of the left and right tails of the absolute returns distribution. In Table 8, the 25th, 50th, 75th and 97.5th percentiles pertaining to Hill tail indexes, across all Monte Carlo simulations, are shown.

**Table 8:** Percentile Values of Hill tail index and Hill index of the left and right tail across all Monte Carlo simulations.

Descriptive statistics	Percentile Value			
	.25	.50	.75	.975
Hill tail index	3.8	3.9	4.1	4.5
Hill right tail index	3.6	3.8	3.96	4.37
Hill left tail index	4	4.2	4.4	5.2

Also for the index of the simulated absolute returns distribution we found values around 4 and the right tail of the distribution is fatter than the left tail.

**Fig 7:**
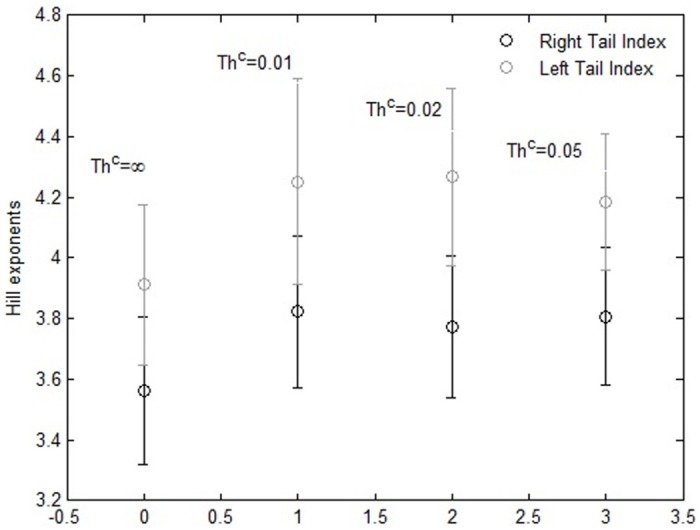
Hill exponents of the right (black) and left (grey) tails of the returns distributions as a function of *Th*^*C*^. The vertical spreads depict the error bars (standard deviation) for the Hill exponent, which are evaluated across 100 runs of the simulations with different random seeds.

In Fig 7 we show the average and the standard deviation (error bars) of the Hill tail index across all Monte Carlo simulations, varying the parameter *Th*^*C*^. Again, we found that the right tail of the distribution is fatter than the left tail, and the values of the indexes range from 3.3 to 4.6. The average value of these indexes increases slightly when Chartists are in the market.

Table 9 shows the 25th, 50th, 75th and 97.5th percentiles pertaining to average and standard deviation of the autocorrelation of raw returns, and those of absolute returns, at time lags between 1 and 20, across all Monte Carlo simulations, varying the parameter *Th*^*C*^. The values reported in Table 9 confirm that the autocorrelation of raw returns is lower than that of absolute returns and that there are not significant differences varying *Th*^*C*^ from 0.01 to ∞. This confirms the presence of volatility clustering also for the simulated price series, irrespective of the presence of Chartists.

**Table 9:** Percentile Values of average and standard deviation of the autocorrelation of raw returns (*avg*_*Ret*_*raw*__ and *std*_*Ret*_*raw*__, respectively) and those of absolute returns (*avg*_*Ret*_*abs*__ and *std*_*Ret*_*abs*__, respectively) across all Monte Carlo simulations, varying the parameter *Th*^*C*^.

*Th*^*C*^	Descriptive statistics	Percentile Value			
		.25	.50	.75	.975
0.01	*avg*_*Ret*_*raw*__	-0.002	0.0004	0.003	0.011
	*avg*_*Ret*_*abs*__	0.075	0.086	0.1	0.12
	*std*_*Ret*_*raw*__	0.04	0.046	0.05	0.06
	*std*_*Ret*_*abs*__	0.035	0.04	0.042	0.05
0.02	*avg*_*Ret*_*raw*__	-0.0032	-0.0008	0.0009	0.01
	*avg*_*Ret*_*abs*__	0.07	0.092	0.097	0.1
	*std*_*Ret*_*raw*__	0.039	0.045	0.05	0.06
	*std*_*Ret*_*abs*__	0.04	0.042	0.044	0.054
0.05	*avg*_*Ret*_*raw*__	-0.004	-0.002	-0.001	0.003
	*avg*_*Ret*_*abs*__	0.08	0.09	0.1	0.11
	*std*_*Ret*_*raw*__	0.042	0.048	0.05	0.06
	*std*_*Ret*_*abs*__	0.032	0.036	0.045	0.057
∞	*avg*_*Ret*_*raw*__	-0.005	-0.002	0.001	0.005
	*avg*_*Ret*_*abs*__	0.08	0.09	0.1	0.11
	*std*_*Ret*_*raw*__	0.042	0.043	0.045	0.053
	*std*_*Ret*_*abs*__	0.032	0.035	0.044	0.06

### Traders’ Statistics

**Fig 8:**
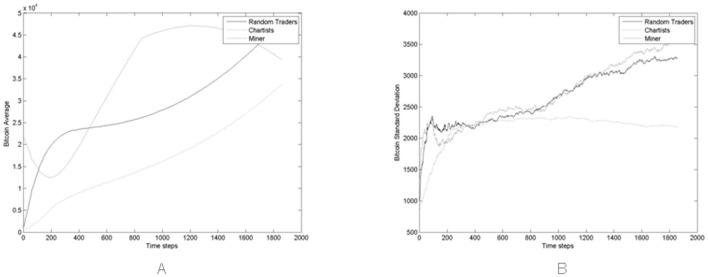
(A) Average and (B) standard deviation of Bitcoin held by all trader populations during the simulation period across all Monte Carlo simulations.

**Fig 9:**
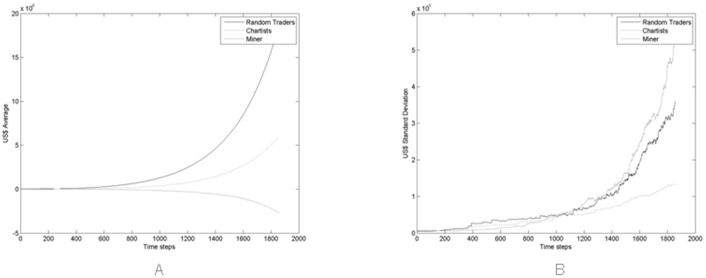
(A) Average and (B) standard deviation of the cash held by all trader populations during the simulation period across all Monte Carlo simulations.

**Fig 10:**
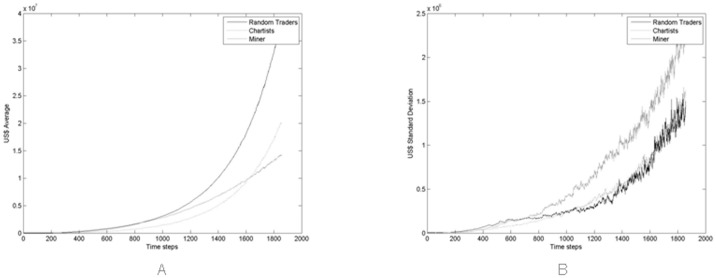
(A) Average and (B) standard deviation of the total wealth of all trader populations during the simulation period across all Monte Carlo simulations.

Figs 8–10 show the average and the standard deviation of the crypto and fiat cash, and of the total wealth, *A*(*t*), of trader populations, averaged across all 100 simulations. These simulations were carried with Miners buying new hardware using an average percentage of 60% of their wealth, that looks to be reasonable (see Fig 12 and discussion thereof).

Fig 10A highlights how Miners represent the richest population of traders in the market, from about step 300 onwards. Note that the standard deviation of the total wealth is much more variable than shown in the former two figures. This is due to the fact that wealth is obtained by multiplying the number of Bitcoins by their price, which is very variable across the various simulations, as shown in Fig 4(B).

**Fig 11:**
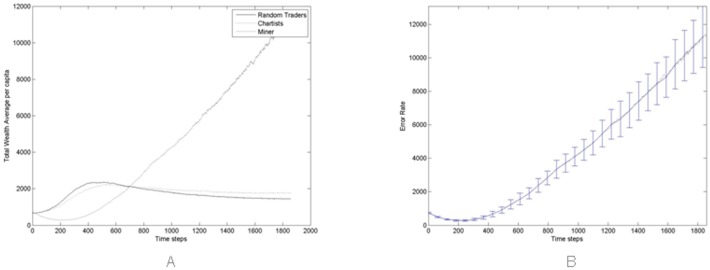
(A) Average across all Monte Carlo simulations of the total wealth per capita of all trader populations for *γ*_1_ = 0.6. (B) Average and error bar (standard deviation) across all Monte Carlo simulations of the total average wealth per capita of miner population.

Fig 11, shows the average of the total wealth per capita of all trader populations, across all 100 Monte Carlo simulations. Miners are again the winners, from about the 700th simulation step onwards, thanks to their ability to mine new Bitcoins. Specifically, thanks to the percentage of cash that Miners allocate to buy new mining hardware, their average wealth per capita—that is about $1,000 at the beginning of the simulation—increases twelve fold to $12,000 at the end. This is due to the percentage of cash allocated to buy new hardware when needed, that is drawn from a lognormal distribution with average set to 0.6 and standard deviation set to 0.15, as already mentioned in the Section *The Agents*.

To assess the stability of this result, we varied the average percentage of the wealth that Miners allocate for buying new hardware, *γ*_1_, to verify how varying this parameter can impact on Miners’ success. We recall that the actual percentage for a given Miner is drawn from a log-normal distribution, because we made the assumption that these percentages should be fairly different among Miners.

**Fig 12:**
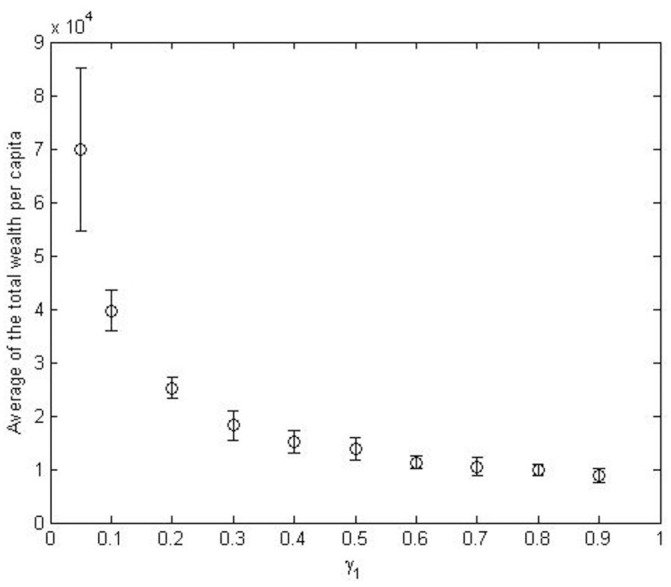
Average of the total wealth per capita for Miners at the end of the simulation period, across all Monte Carlo simulations for increasing values of the average of *γ*_1_.

Fig 12 shows the average and the standard deviation (error bars) of the total wealth per capita for Miners, at the end of the simulation period, for increasing values of the average of *γ*_1_. It is apparent that Miners’ gains are inversely proportional to *γ*_1_, so the general strategy of devoting more money to buy hardware looks not successful for Miners. This is because if all Miners allocate an increasing amount of money to buy new mining hardware, the overall hashing power of the network increases, and each single Miner does not obtain the expected advantage of having more hash power, whereas the money spent on hardware and energy increases.

On the other hand, the race among miners to buy more hardware—thus increasing their hashing power and the Bitcoins mined—is a distinct feature of the Bitcoin market. We stipulate that it is not reasonable that Miners allocate over 60% of their wealth every few months to buy hardware and pay electricity bills. Fig 12 shows that it is precisely the value *γ*_1_ = 0.6 when Miners’ total wealth per capita at the end of the simulation stabilizes. We therefore used this value for our simulations.

We also studied the total wealth average per capita for all trader populations varing *σ*^*id*^, the standard deviation in the definition of the time when the Miners decide to buy or divest hardware units. Our analysis does not highlight significant difference varying *σ*^*id*^. Further results about the impact of these two parameters on the simulation results is presented in *Appendix E*, in S1 Appendix.

Having found that Miners’ wealth decreases when too much of it is used to buy new hardware, we studied if increasing the money spent in mining hardware would be a successful strategy for single Miners, when most other Miners do not follow it.

**Fig 13:**
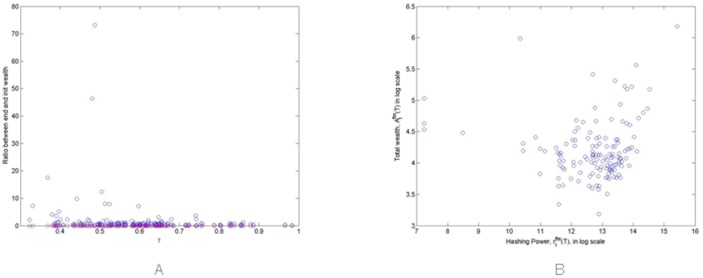
Scatterplots of (A) increase in wealth of single Miners versus their average wealth percentage used to buy mining hardware, and (B) total wealth of Miners versus their hashing power at the end of the simulation.

Fig 13A shows the ratio of initial Miners’ total wealth computed at the end and at the beginning of a single simulation, $\frac{A_{i}^{f_{m}}\left(T\right)}{A_{i}^{f_{m}}\left(0\right)}$, versus their actual value of *γ*_*i*_, that is their propensity to spend money to buy mining hardware. In this simulation we set <*γ* > = 0.6. The computed correlation coefficients is equal to -0.3, so it looks like that there is no meaningful correlation between mining success and the propension to invest in hardware. In Fig 13A we can see that all the three most successful Miners, able to increase their wealth from 18 fold to 74 fold, have values of *γ*_*i*_ less than 0.5, compared to the average <*γ* > = 0.6.

We also found that the total wealth of Miners at the end of the simulation, $A_{i}^{f_{m}}\left(T\right)$, is correlated with their hashing capability $r_{i}^{f_{m}}\left(T\right)$, as shown in Fig 13B, the correlation coefficient being equal to 0.81. This result is not unexpected because wealthy Miners can buy more hardware, that in turn helps them to increase their mined Bitcoins.

**Fig 14:**
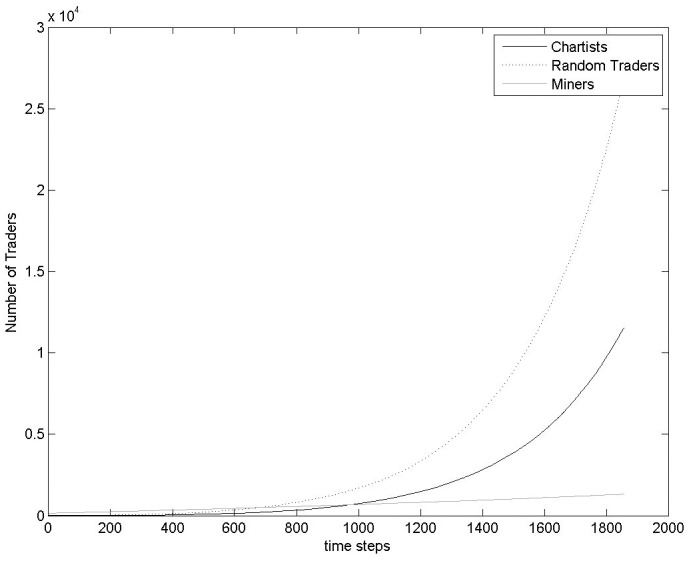
Number of Traders over time. These values are the same across all Monte Carlo simulations.

Fig 14 shows the number of traders belonging to each population of traders, Chartists, Random traders and Miners. According to the definition of the probability of a trader to belong to a specific trader population, these numbers are the same across all 100 Monte Carlo simulations (see *Appendix D*, in S1 Appendix).

### Statistics Related to Hashing Power and Power Consumption

**Fig 15:**
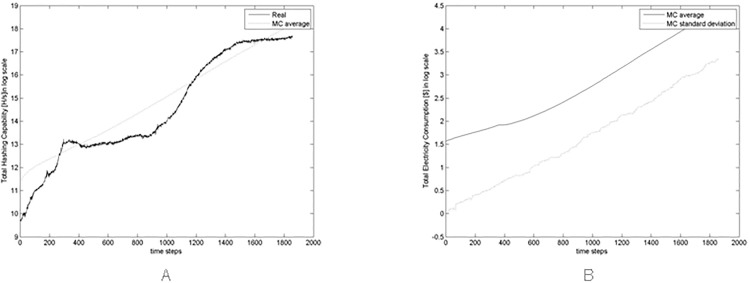
(A) Comparison between real hashing capability and average of the simulated hashing capability across all Monte Carlo simulations (multiplied by 100) in log scale, and (B) average and standard deviation of the total expenses in electricity across all Monte Carlo simulations in log scale.

Fig 15A shows the average hashing capability of the whole network in the simulated market across all Monte Carlo simulations and the hashing capability in the real market. These quantities are both expressed in log scale. Note that the simulated hashing capability is multiplied by 100, that is the scaling factor of our simulations, which have 1/100 th of the real number of Bitcoin traders and miners.

The simulated hash rate does not follow the upward trend of the Bitcoin price at about the 1200th time step that is due to exogenous causes (the steep price increase at the end of 2013), that is obviously not present in our simulations. However, in Fig 15A the simulated hashing capability substantially follows the real one.

**Fig 16:**
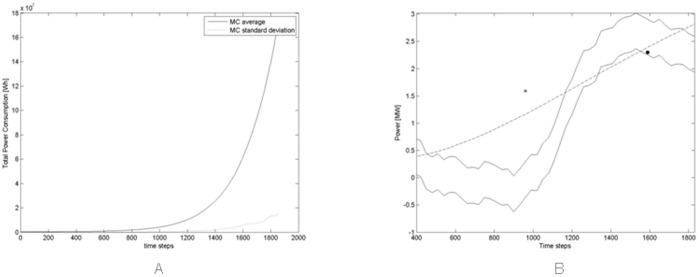
(A) Average and standard deviation of the power consumption across all Monte Carlo simulations. (B) Estimated minimum and maximum power consumption of the real Bitcoin Mining Network (solid lines), and average of the power consumption across all Monte Carlo simulations, multiplied by 100, the scaling factor of our simulations (dashed line). For the meaning of the diamond and circle, see text.

Fig 16A shows the average and standard deviation of the power consumption across all Monte Carlo simulations. Fig 16B shows an estimated minimum and maximum power consumption of the Bitcoin mining network, together with the average of the power consumption of Fig 16(a), in logarithmic scale. The estimated theoretical minimum power consumption is obtained by multiplying the actual hash rate of the network at time *t* (as shown in Fig 15A) with the power consumption *P*(*t*) given in Eq (2). This would mean that the entire hashing capability of Miners is obtained using the most recent hardware. The estimated theoretical maximum power consumption is obtained by multiplying the actual hash rate of the network with the power consumption *P*(*t* − 360), referring to one year before. This would mean that the entire hashing capability of Miners is obtained with one year old hardware, and thus less efficient. The estimated obsolescence of mining hardware is between six months and one year, so the period of one year should give a reliable maximum value for power consumption.

The simulation results, averaged on 100 simulations, show a much more regular trend, steadily increasing with time—which is natural due to the absence of external perturbations on the model. However, the power consumption value is of the same order of magnitude as the “real” case. Also in this case the simulated consumption shown in Fig 16B is multiplied by 100, that is the scaling factor of our simulations.

Fig 16B also shows a diamond, at time step corresponding to April 2013, with a value of 38.8 MW. This value has been taken by Courtois et al, who write in work [30]:

In April 2013 it was estimated that Bitcoin miners already used about 982 Megawatt hours every day. At that time the hash rate was about 60 Tera Hash/s. (Refer to article by Gimein Mark “Virtual Bitcoin Mining Is a Real-World Environmental Disaster”, 13 April 2013 published on web site www.Bloomberg.com).

In fact, the hash rate quoted is correct, but the consumption value looks overestimated of one order of magnitude, even with respect to our maximum power consumption limit. We believe this is due to the fact that the authors still referred to FPGA consumption rates, not fully appreciating how quickly the ASIC adoption had spread among the miners.

As of 2015, the combined electricity consumption was estimated equal to 1.46 Tera Wh per year, which corresponds to about 167 MW (see article “The magic of mining”, published on web site www.economist.com on 13 January 2015). This value is reported in Fig 16B as a circle. This time, the value is slightly underestimated, being on the lower edge of the power consumption estimate, and is practically coincident with the average value of our simulations.

**Fig 17:**
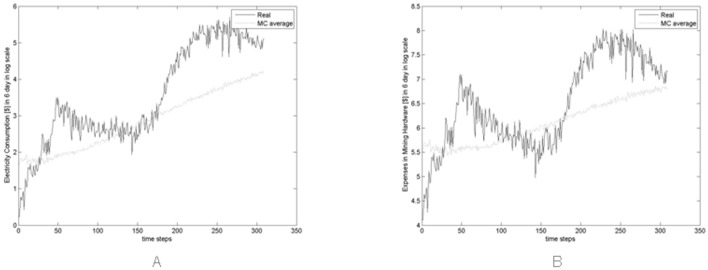
(A) Real expenses and average expenses in electricity across all Monte Carlo simulations. (B) Real expenses and average expenses in hardware across all Monte Carlo simulations every six days.

Fig 17 show an estimate of the total expenses incurred every six days in electricity Fig 17A and in hardware Fig 17B for the new hardware bought each day in the real and simulated market. Note that also in this case the values of the simulated expenses are averaged across all Monte Carlo simulations.

These expenses are the expenses incurred in six days by Miners, hence they are obtained by summing the daily expenses related to the new hardware bought. We assumed that the new hardware bought each day in the real (simulated) market is equal to the difference between the real (simulated) hash rate in *t* and the real (simulated) hash rate in *t* − 1. In other words, we assumed that the new hardware bought each day is the additional hashing capability acquired each day.

The daily expenses in hardware were computed by multiplying the additional hashing capability acquired each day by the cost related to the additional hashing capability. This cost is defined as $\frac{1}{R(t)}$, where *R*(*t*) is the average hash rate per US$ spent on hardware as in Eq (1). Instead, the daily expenses in electricity were computed by multiplying the additional hashing capability acquired each day by the electricity cost, computed as in in Eq (2) and related to the additional hashing capability.

For both these expenses, contrary to what happens to the respective real quantities, the simulated quantities do not follow the upward trend of the price, due to the constant investment rate in mining hardware.

**Fig 18:**
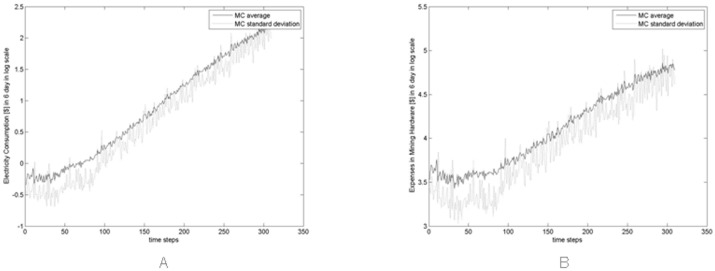
Average and standard deviation of the expenses in electricity (A) and of the expenses in new hardware (B) across all Monte simulations.

Fig 18A and 18B show the average and standard deviation, across all Monte simulations, of the expenses incurred every six days in electricity and in new hardware respectively, showing the level of the variation across the simulations.

Remembering that our model sizes the artificial market at about 1/100 of the real market and that the number of traders, their cash and their trading probabilities are rough estimates of the real ones, the simulated market outputs can be considered reasonably close to the real ones.

## Conclusions

In this work, we propose a heterogeneous agent model of the Bitcoin market with the aim to study and analyze the mining process and the Bitcoin market starting from September 1st, 2010, the approximate date when miners started to buy mining hardware to mine Bitcoins, for five years.

The proposed model simulates the mining process and the Bitcoin transactions, by implementing a mechanism for the formation of the Bitcoin price, and specific behaviors for each typology of trader. It includes different trading strategies, an initial distribution of wealth following Pareto’s law, a realistic trading and price clearing mechanism based on an order book, the increase with time of the total number of Bitcoins due to mining, and the arrival of new traders interested in Bitcoins.

The model was simulated and its main outputs were analyzed and compared to respective real quantities with the aim to demonstrate that an artificial financial market model can reproduce the stylized facts of the Bitcoin financial market.

The main result of the model is the fact that some key stylized facts of Bitcoin real price series and of Bitcoin market are very well reproduced. Specifically, the model reproduces quite well the unit-root property of the price series, the fat tail phenomenon, the volatility clustering of the price returns, the generation of Bitcoins, the hashing capability, the power consumption, and the hardware and electricity expenses incurred by Miners.

The proposed model is fairly complex. It is intrinsically stochastic and of course it includes endogenous mechanisms affecting the market dynamics. We performed some analysis of the sensitivity of the model to some key parameters, finding that the “herding” effect of Chartists, when a price trend is established, does not play a key role in the distribution of the price returns, and hence in the reproduction of the fat tail phenomenon that stem from the market microstructure rather than from sophisticated behavioral assumptions, as in the work by Licalzi et al. [25]; the Chartist behavior does not even affect other stylized facts, like the volatility clustering and unit-root property; the total wealth per capita for Miners varies with the average percentage value of their wealth allocated for buying new hardware, keeping substantially unchanged both their average hashing capability, and their average expenses in electricity, computed across all Monte Carlo simulations; finally, the heterogeneity in the fiat and crypto cash of the traders emerges endogenously also when traders start from the same initial wealth.

Future research will be devoted to studying the mechanisms affecting the model dynamics in deeper detail. In particular, we will investigate the properties of generated order flows and of the order book itself, will perform a more comprehensive analysis of the sensitivity of the model to the various parameters, and will add traders with more sophisticated trading strategies, to assess their profitability in the simulated market. In addition, since the calibration of our model is based on very few specific real data, and on many assumptions aiming to derive the needed data from indirect real data, we plan to perform a deeper analysis of the Blockchain, and to gather financial data from existing exchanges, in order to extract specific information needed for a better calibration of our model.

## Supporting Information

S1 Appendix Our appendices A, B, C, D and E are in the file “S1_Appendix.pdf”. (PDF)

S1 Data The file “S1_Data.pdf” contains the value of real Bitcoin price from September 1st, 2010 to September 30th, 2015. Note that data in the file “S1 Data.txt” is carriage return–separated.
(PDF)

S2 Data The file “S2_Data.pdf” contains the value of the hash rate in the real Bitcoin network from September 1st, 2010 to September 30th, 2015. Note that data in the file “S2 Data.txt” is carriage return–separated.
(PDF)

S3 Data The file “S3_Data.pdf” contains the value of the transaction number in the real Bitcoin network from September 1st, 2010 to September 30th, 2015. Note that data in the file “S3 Data.txt” is carriage return–separated.
(PDF)

S4 Data The file “S4_Data.pdf” contains the value of simulated Bitcoin price from September 1st, 2010 to September 30th, 2015. Note that data in the file “S4 Data.txt” is carriage return–separated.
(PDF)

S5 Data The file “S5_Data.pdf” contains data about traders. Exactly data stored in this file is the following.
crypto cash of the Random traders,fiat cash of the Random traders,crypto cash of the Chartists,fiat cash of the Chartists,crypto cash of Miners,fiat cash of Miners,average of the total hashing capability in the network across all traders,average of the total energy consumption in the network across all traders,total hashing capability in the network,total energy consumption in the network,average of the bitcoin mined in the network across all miners.
Note that data in “S5_Data.pdf” is stored as follows: to each variable corresponds a row of data separated by Tab characters, that are the values of the variable at each simulation step.each row of data, associated to a given variable, ends in a carriage return. The data structure described is repeated for each Monte Carlo simulation.
(PDF)

S6 Data The file “S6_Data.pdf” contains the number of Chartists in the simulated market from September 1st, 2010 to September 30th, 2015 for all Monte Carlo simulations. Note that data in the file “S6 Data.txt” is carriage return–separated.
(PDF)

S7 Data The file “S7_Data.pdf” contains the number of Miners in the simulated market from September 1st, 2010 to September 30th, 2015 for all Monte Carlo simulations. Note that data in the file “S7 Data.txt” is carriage return–separated.
(PDF)

S8 Data The file “S8_Data.pdf” contains the number of Random traders in the simulated market from September 1st, 2010 to September 30th, 2015 for all Monte Carlo simulations. Note that data in the file “S8 Data.txt” is carriage return–separated.
(PDF)
