# Supplementary material for: Modeling and Simulation of the Economics of Mining in the Bitcoin Market
Source: PLoS One. 2016 Oct 21;11(10):e0164603. doi: 10.1371/journal.pone.0164603 (PMC5074464; doi:10.1371/journal.pone.0164603)
Supplement: S1 Appendix — (PDF) [file pone.0164603.s001.pdf]

Appendix

A. Trader Wealth Endowment

The distributions of cash and Bitcoins, for traders in the market at initial time, follow a power-law with exponent α set equal to 1, a value yielding the distribution known as Zipf's law [1]. This is the same assumption made in other works on artificial financial markets [2, 3]. We assumed that 40% of the total initial crypto cash is not involved in the trading activity, given that initial Miners hoarded a fraction of their Bitcoins (see web site http://www.coindesk.com/mit-report-bitcoin-more-likely-spent-hoarded/.).

To create Zipf's distribution, we used the ranking property of the power-law [4]. If the total number of traders is N_t and the number of Bitcoins owned by them, b_i, follows a Pareto law with exponent $\alpha = 1$, it is well-known from the Harmonic-series theory that the total number of Bitcoins $B_T = b_1 ln(N_t) + \gamma$, where γ is the Euler-Mascheroni constant and b_1 is the number of Bitcoins owned by the richest trader. The number of Bitcoins owned by i-th trader is consequently: $\frac{b_1}{i}$. We set the nominal cash of each initial trader equal to five times the nominal value of their crypto cash.

A similar approach was followed to set the wealth of traders who enter the simulation at $t > 0$, but in this case the traders are only endowed with cash. In this case, we had no specific data to calibrate the wealth of these traders. We stipulated that the cash, c_1^s, of the richest trader is about five times the cash owned by the richest initial trader, and that the exponent of the Pareto law is in this case $\alpha = 0.6$. Overall, we performed various simulations varying these parameters, with no significant impact on the results.

The set of "new" traders are generated before the simulation starts. When new traders are needed to enter the simulation, they are chosen randomly in this set.

In general, in financial artificial agent-based models, the heterogeneity in the fiat cash and assets of the traders often emerges endogenously also when traders start from the same initial wealth. We performed several simulations studying how the fiat and crypto cash of each population of traders evolve over time starting from the same initial values. Specifically, we set the crypto cash of the initial traders equal to 87.28 Bitcoins, that is equal to the ratio between the total initial crypto cash in the market and the total initial number of traders. We set the fiat cash of both initial traders and new traders equal to five times the crypto cash, and hence equal to 436.39$. In Table 1 we report the 25th, 50th, 75th and 97.5th percentiles pertaining to the crypto and fiat cash per capita, at the end of the simulation period. They are computed considering each specific population of traders, and the population of all traders.

The values show that the heterogeneity per capita in the fiat and crypto cash of the traders emerges endogenously also when traders start from the same initial wealth. Miners show the highest heterogeneity, followed by Random traders. Chartists' wealth, on the contrary, shows a more homogeneous behavior. In addition, this analysis showed that all simulation results are substantially unchanged with respect to those described in Section *Simulation Results* when traders start from the same initial wealth.

B. Number of Traders

One of the most attractive property of Bitcoin is to provide quasi-anonymous transactions. So, knowing the number of traders in the real market is very difficult. The

Table 1. Percentile Values of crypto and fiat (in brackets) cash per capita owned by each type of trader, and by all traders, in one typical simulation run at the end of the simulation period, when they start from the same initial values of fiat and crypto cash.

Traders' Population	Percentile Value			
	.25	.50	.75	.975
All traders	0.72 (312)	1.2 (470)	2 (601)	13 (1,548)
Miners	3.3 (-3,814)	14 (-684)	64 (-197)	128 (6,773)
Random traders	0.7 (306)	1.2 (484)	1.8 (667)	5.1 (1,788)
Chartists	0.7 (369)	1.1 (468)	2 (540)	12 (624)

Bitcoin addresses used for the transactions are known, but a user can have, and typically has, more than one address.

At the moment of writing (last quarter of 2015) we had three figures:

1. the massive and transparent ledger of every Bitcoin transaction was generated starting since January 3, 2009, by the inventor of the Bitcoin system himself, Nakamoto and so in January 2009 there was only one person owning Bitcoins;
2. "according to rough estimates, 280,000 people owned Bitcoins at the end of 2013" (http://www.whoishostingthis.com/blog/2014/03/03/who-owns-all-the-bitcoins/);
3. on April 22nd, 2014 the total number of holders was estimated equal to 1.0 million (https://Bitcointalk.org/index.php?topic=316297.0).

In addition to these figures, we have other data related to the period between January 2009 and September 2010. These data were extracted from an analysis on the daily number of downloads of the official Bitcoin software client from the SourceForge platform (http://sourceforge.net/projects/bitcoin).

In the period just mentioned, the Bitcoin network began to spread and Bitcoin had no monetary value. So, for this period we assumed the number of downloads of the official Bitcoin software client to be equal to the number of traders in the market. We made the assumption that a person who downloaded the Bitcoin software was mainly interested to use it to mine Bitcoins. So, we extracted two figures from these data: the total number of downloads on May 1st, 2010, equal to 2,769, and the total number of downloads on September 1st, 2010, equal to 30,589, and we set the number of downloads equal to the number of traders.

We then computed the number of traders in the market fitting the curve N_T through the five figures available:

1. 1 person owned Bitcoins in January 2009. He was Satoshi Nakamoto;
2. 2769 people had downloaded Bitcoin mining software in May 2010;
3. 30,589 people had downloaded Bitcoin mining software in September 2010;
4. 280,000 people owned Bitcoins at the end of 2013;
5. 1,000,000 people owned Bitcoins in April 2014.

The fitting curve of the number of traders N_T is defined by using a general exponential model:

$$N_T(t) = a * e^{(b*(608+t))} \quad (1)$$

where $a = 2624$, $b = 0.002971$.

Fig 1A shows the fitting curve and how the number of traders increases over time.

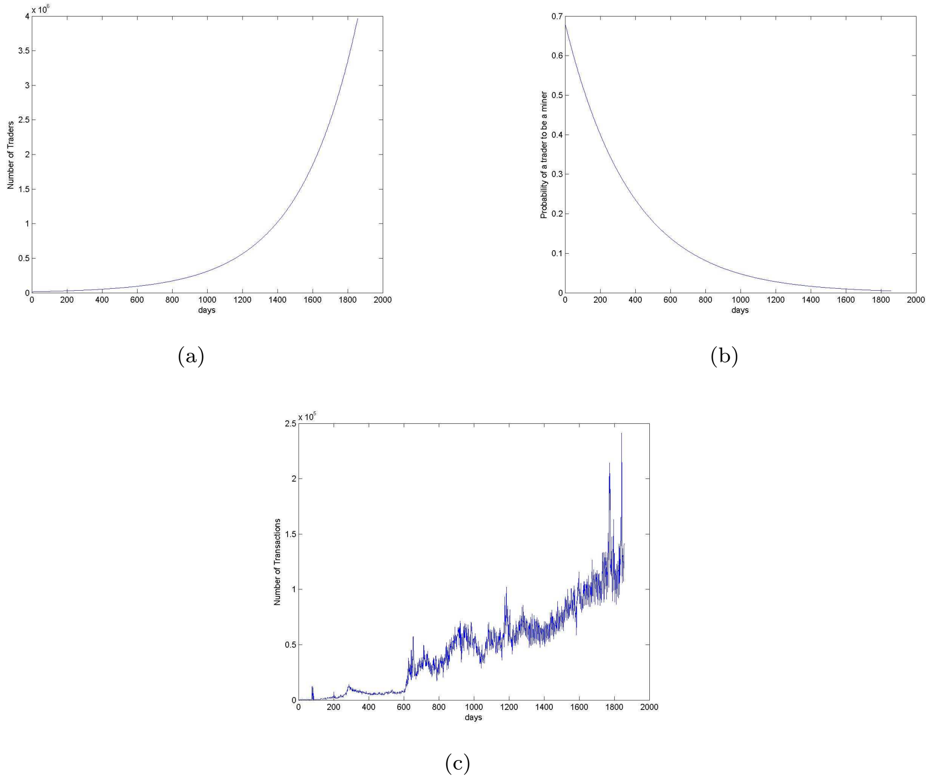

Figure 1. Fitting curve (A) of $N_T(x)$, (B) of the probability of a trader to be a Miner, and (C) number of real Bitcoin transactions.

C. Active Traders

As mentioned in subsection *The agents*, only a given percentage of traders is active in the market, and hence enabled to issue orders. To compute this percentage we made some assumptions starting from the work [5] from which we extracted Table 2, which shows the distribution of the transactions number per entity (*Entity* means the common owner of multiple Bitcoin addresses) and per address on a period between January 3rd, 2009 and May $13th$ 2012.

Table 2. *The distribution of the number of transactions per entity and per address.*

Number of Transactions (n)	Number of entities	Number of addresses
$1 \leq n < 2$	557,783	495,773
$2 \leq n < 4$	1,615,899	2,197,836
$4 \leq n < 10$	222,433	780,433
$10 \leq n < 100$	55,875	228,275
$100 \leq n < 1,000$	8,464	26,789
$1,000 \leq n < 5,000$	287	1,032
$5,000 \leq n < 10,000$	35	51
$10,000 \leq n < 100,000$	32	24
$100,000 \leq n < 500,000$	7	3
$n \geq 500,000$	1	2

By analyzing Table 2, we can observe that 97.37% of all entities made fewer than 10 transactions each, 2.27% of all entities made a number of transactions ranging from 10 to 100, 0.34 % of all entities made a number of transactions ranging from 100 to 1,000, and the remaining 0.02 % made a number of transactions higher than 1,000.

According to the insights coming from this work, we neglected the entities having fewer than 10 transactions each and those with more than 1,000 transactions each. This was done hypothesizing that the former, typically involving a small number of Bitcoins, refer to entities who made transactions by chance "only to use" this new coin, whereas the latter, involving a very high number of transactions, are probably not linked to single traders, but are the addresses of exchange sites, or of retailers accepting Bitcoins.

We considered the remaining entities, that is the entities with a number of transactions in the range from 10 to 1,000. They issued orders with a period ranging from about one day to about 122 days. Since these data are computed on 1227 days, the daily trading probability of an entity ranges from 0.008% to 1%.

We set the values of trading probabilities for Random traders and Chartists in this range. Specifically, we assumed that Random traders were active with a probability $p_R^t = 0.1$, whereas the Chartists were active with a probability $p_C^t = 0.5$. This because the interest of Chartists in purchasing or selling Bitcoins is higher than that of Random traders. Random traders issue orders to satisfy their needs, whereas Chartists issue orders for profit reasons, study carefully the price variation over time and are readier to place orders. Note that active Chartists actually place orders only if the price variation is above a given threshold.

D. Probability of a trader to belong to a specific traders' population

At first, users in the Bitcoin network were mainly miners. When the Bitcoin network started to grow and Bitcoins started to acquire a monetary value, new users entered the network. Most of these users did not mine, but simply traded Bitcoins. They are represented in the model as Random traders and Chartists.

Since the percentages of the different trader populations is not known, we carried out an analysis of the Bitcoin Blockchain to compute the probability of a trader to be a Miner. We analyzed the Blockchain up to May 1st, 2010 and then up to September 1st, 2010. Each block in the Blockchain contains a list of validated transactions. Each transaction has in input all the addresses containing the amount of Bitcoins to transfer, and in output all the addresses that receive the Bitcoins. Users can use multiple addresses.

We assumed, as in [6], that the input addresses that send Bitcoins to the same output address must belong to a unique owner, since it is necessary to know the private keys of all input addresses in order to proceed with the transaction.

In addition, we considered the input addresses that transfer more than 20 Bitcoins owned by the same owner of the corresponding outputs. In fact, in the period from May 1st, 2010, to September 1st, 2010 Bitcoins had no monetary value, and so it is acceptable to consider that miners exchanged only small amounts of Bitcoins to test the operation of the network. Of course, this last assumption can be valid only for the period under study.

With these assumptions, we found 46,005 unique addresses on May 1st, 2010 and 82,294 unique addresses on September 1st, 2010. With further analysis, we found that on May 1st, 2010, 43,389 out of 46,005 addresses were addresses that mined, and on September 1st, 2010, 55,974 out of 82,294 addresses were addresses that mined, so we computed the probability of a user to be a Miner. This probability is equal to 0.94% on

May 1st, 2010 and equal to 0.68% on September 1st, 2010.

Using these two figures we computed a fitting curve of the probability of a user to be a Miner p_M. Again, it is defined by using a general exponential model:

$$p_M(t) = a * e^{(b*t)} \tag{2}$$

where $a = 0.9425$, $b = -0.002654$.

Fig 1B shows the fitting curve and how this probability decreases over time. We see that the probability of a trader to be a Miner decreases over time, going from about 0.38 to less than 0.01. Of course, defining this probability using a fitting curve computed from only two points is questionable. In the following, we made some considerations to validate the adoption of this curve.

At first, the number of Bitcoin transactions was low because in the market there were mainly miners. Over time, as Bitcoin was acquiring monetary value, the number of users interested in exchanging Bitcoins increased. So, while the percentage of miners in the market was decreasing, also due to the increasing difficulty to mine Bitcoins, the percentage of Random traders p_R and Chartists p_C greatly increased, according to the growth of the number of transactions, which slowly rose until a peak on May 2012 and then at the end of the 2013 (see Fig 1C).

We assumed that the Random traders to Chartist ratio is 7/3, meaning that 30% of traders who are not Miners are speculators, whereas the remaining 70% are non-speculative investors. These figures are consistent with recent data obtained for the foreign exchange market [7]. The probabilities of a non-Miner to be a Random trader or Chartist, p_R and p_C respectively, are defined as a function of p_M, respectively as:

$$p_R = 0.7(1 - p_M) \tag{3}$$

and

$$p_C = 0.3(1 - p_M) \tag{4}$$

With these probabilities, we have a number of Miners equal to about 1000 at the end of the simulations, corresponding to 100,000 miners in the real world. This is in agreement with what an Australian Bitcoin miner, Andrew Geyl, estimated (see web site http://bravenewcoin.com/news/number-of-bitcoin-miners-far-higher-than-popular-estimates/).

E. Sensitivity Analysis: Other Results

In the following we present other results about the sensitivity of the model to the parameters γ_1 and σ^{id}. Remember that γ_1 is the percentage of cash allocated to buy new hardware by Miners, and σ^{id} is the standard deviation entering in the definition of the instant in which the Miners decide to buy or divest hardware units. In Section *Traders' Statistics* we showed how the variations of these parameters affect the total wealth average per capita for all trader populations at the beginning and at the end of the simulation period. In this section we give further insights on the effects of these parameters on simulation results.

Figs 2A and 3A show a comparison between real hashing capability and average of the simulated hashing capability across all Monte Carlo simulations in log scale, for $\gamma_1 = 0.1$ and for $\gamma_1 = 0.9$, respectively. The average of the simulated hashing capability shown in Figs 2A and 3A is multiplied by 100, that is the scaling factor of our simulations. The figures show that, despite a substantial increase in the percentage of Miners' wealth devoted to buy new hardware, there is only a small increase of the simulated total hashing capability. This is due probably to a self-regulating mechanism.

If Miners devote a small percentage of their wealth to buy hardware, this wealth tend to increase quickly (due to mining), and the actual money devoted to buy hardware actually increases. If, on the other hand, Miners spend a high percentage of their wealth to buy hardware, soon their wealth is much lower, and a lot of it is wasted to pay the electricity bill. In the end, the total amount of hardware bought does not differ too much between the two cases.

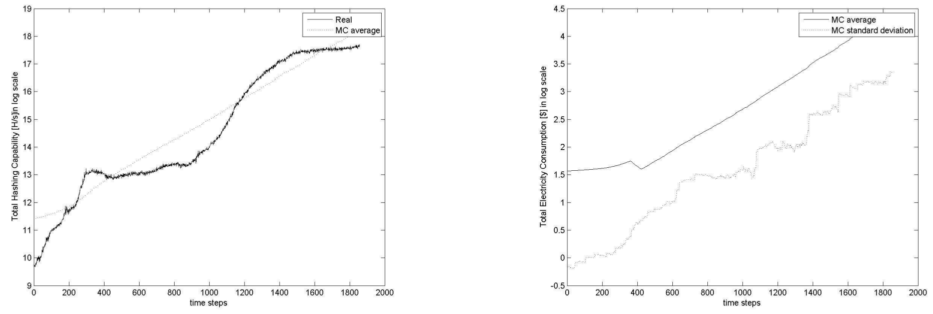

(a) (b)

Figure 2. (A) Comparison between real hashing capability and average of the simulated hashing capability across all Monte Carlo simulations in log scale, and (B) average and standard deviation of the total expenses in electricity across all Monte Carlo simulations in log scale, for $\gamma_1 = 0.1$ and $\sigma^{id} = 6$.

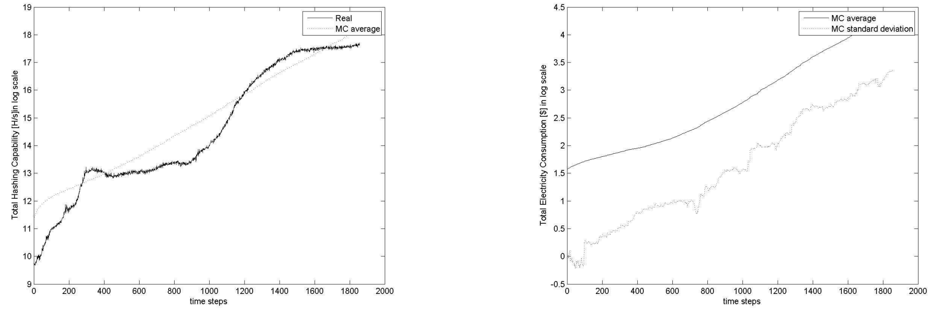

(a) (b)

Figure 3. (A) Comparison between real hashing capability and average of the simulated hashing capability across all Monte Carlo simulations in log scale, and (B) average and standard deviation of the total expenses in electricity across all Monte Carlo simulations in log scale, for $\gamma_1 = 0.9$ and $\sigma^{id} = 6$.

This conclusion is confirmed by the analysis of the impact of varying parameter $\gamma_1$on the estimate of the expenses incurred every six days in electricity and in hardware, for the new hardware bought each day, reported in Figs 4–5.

When the average percentage of wealth devoted by Miners to buy hardware is low (Fig 4, $\gamma_1 = 0.1$), the real expenses in both electricity and hardware start low, but quickly reach values comparable to when this percentage is high (Fig 4, $\gamma_1 = 0.9$).

As regards the parameter σ^{id}, Table 3 shows the average and the standard deviation of the average of the total wealth per capita for Miners across all Monte Carlo simulations, for increasing values of σ^{id} keeping fixed the value of γ_1 to 0.6, that is the

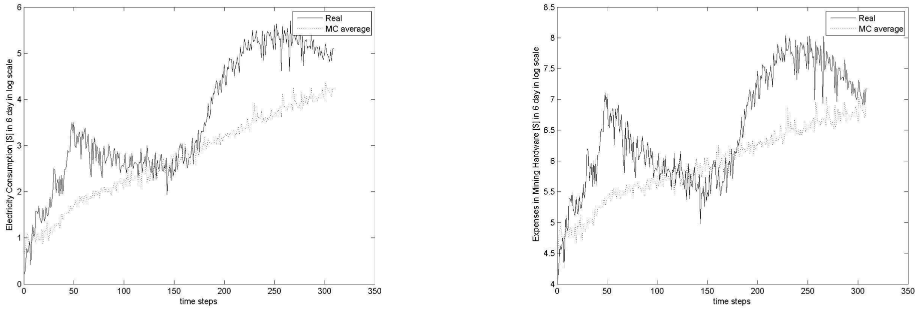

(a) (b)

Figure 4. (A) Real expenses in electricity and average of the simulated expenses in electricity. (B) Real expenses in hardware and average of the simulated expenses in hardware across all Monte Carlo simulations, computed every six days, for $\gamma_1 = 0.1$ and $\sigma^{id} = 6$.

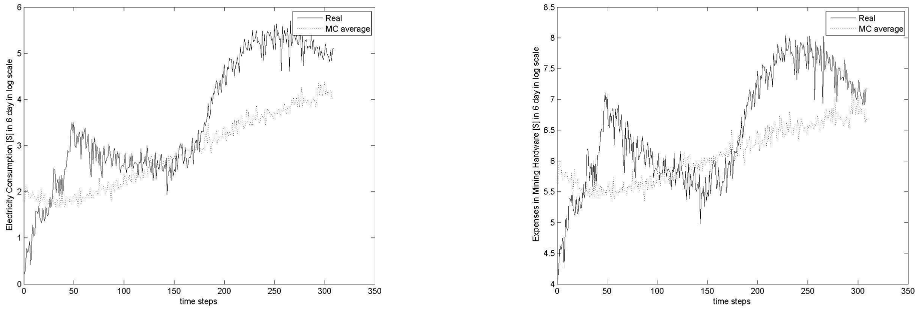

(a) (b)

Figure 5. (A)Real expenses in electricity and average of the simulated expenses in electricity (B) Real expenses in hardware and average of the simulated expenses in hardware across all Monte Carlo simulations, computed every six days, for $\gamma_1 = 0.9$ and $\sigma^{id} = 6$.

value at which the wealth per capita at the end of the simulation becomes relatively stable, after a steep decrease. These results do not highlight any significant difference varying of σ^{id}.

We carried on other analyses, wich highlighted that this parameter does not impact on the average of the simulated hashing capability across all Monte Carlo simulations, and on the estimate of the expenses incurred every six days in electricity and in hardware by miners for the new hardware bought each day.

References

1. Newman M.E.J. Power laws, Pareto distributions and Zipf's law, Contemporary Physics. 2005; 46(5): 323-351.

2. Raberto M, Cincotti S, Focardi S, Marchesi M. Agent-based simulation of a financial market. Physica A. 2001; 299(1): 319–327.

Table 3. Average and standard deviation of the average of the total wealth per capita for Miners, for decreasing values of the average of σ^{id}, across all Monte Carlo simulations.

σ^{id}	Descriptive statistics	t=0	t=T
10	*avg*	739.81	12,117
	std	31.19	1,658
8	*avg*	759.56	11,083
	std	11.43	1,070
6	*avg*	724.11	11,239
	std	46.37	1,574
4	*avg*	748.68	10,907
	std	20.56	1,948
2	*avg*	713.44	12,263
	std	62.45	2,482

3. Raberto M, Cincotti S, Focardi S, Marchesi M. Traders' Long-Run Wealth in an Artificial Financial Market. Computational Economics, Kluwer. 2003; 22(3): 255–272.

4. Takayasu, H. Fractals in the Physical Sciences. John Wiley and Sons, New York.1990.

5. Ron D, Shamir A. Quantitative Analysis of the Full Bitcoin Transaction Graph. Financial Cryptography and Data Security Volume 7859 of the series Lecture Notes in Computer Science. 2013; 6–24.

6. Garcia D, Tessone C.J, Mavrodiev P, Perony N. Feedback cycles between socio-economic signals in the Bitcoin economy The digital traces of bubbles: feedback cycles between socio-economic signals in the Bitcoin economy. CoRR. 2014; 1408.1494.

7. Dick C. D, MenkhoffL. Exchange rate expectations of chartists and fundamentalists. Journal of Economic Dynamics & Contro2013; 37: 1362–1383.
