# Supplementary material for: Modeling and Simulation of the Economics of Mining in the Bitcoin Market
Source: PLoS One. 2016 Oct 21;11(10):e0164603. doi: 10.1371/journal.pone.0164603 (PMC5074464; doi:10.1371/journal.pone.0164603)
Supplement: S1 Data — Note that data in the file “S1 Data.txt” is carriage return–separated. (PDF) [file pone.0164603.s002.pdf]

The file "S1 Data.txt" contains the value of Bitcoin price from September 1st, 2010 to September 30th, 2015.

Note that data in the file "S1 Data.txt" is carriage return--separated.
