# Supplementary material for: Modeling and Simulation of the Economics of Mining in the Bitcoin Market
Source: PLoS One. 2016 Oct 21;11(10):e0164603. doi: 10.1371/journal.pone.0164603 (PMC5074464; doi:10.1371/journal.pone.0164603)
Supplement: S2 Data — Note that data in the file “S2 Data.txt” is carriage return–separated. (PDF) [file pone.0164603.s003.pdf]

The file "S2 Data.txt" contains the value of the hash rate in the real Bitcoin network from September 1st, 2010 to September 30th, 2015.

Note that data in the file "S2 Data.txt" is carriage return--separated.
