# Supplementary material for: Modeling and Simulation of the Economics of Mining in the Bitcoin Market
Source: PLoS One. 2016 Oct 21;11(10):e0164603. doi: 10.1371/journal.pone.0164603 (PMC5074464; doi:10.1371/journal.pone.0164603)
Supplement: S5 Data — Exactly data stored in this file is the following. crypto cash of the Random traders,fiat cash of the Random traders,crypto cash of the Chartists,fiat cash of the Chartists,crypto cash of Miners,fiat cash of Miners,average of the total hashing capability in the network across all traders,average of the total energy consumption in the network across all traders,total hashing capability in the network,total energy consumption in the network,average of the bitcoin mined in the network across all miners. Note that data in “S5_Data.pdf” is stored as follows: to each variable corresponds a row of data separated by Tab characters, that are the values of the variable at each simulation step.each row of data, associated to a given variable, ends in a carriage return. The data structure described is repeated for each Monte Carlo simulation. (PDF) [file pone.0164603.s006.pdf]

The file "S5 Data.txt" contains data about traders. Exactly data stored in this file is the following:

- crypto cash of the Random traders,
- fiat cash of the Random traders,
- crypto cash of the Chartists,
- fiat cash of the Chartists,
- crypto cash of Miners,
- fiat cash of Miners,
- average of the total hashing capability in the network across all traders,
- average of the total energy consumption in the network across all traders,
- total hashing capability in the network,
- total energy consumption in the network,
- average of the bitcoin mined in the network across all miners.

Note that data in "S5 Data.txt" is stored as follows:

- to each variable corresponds a row of data separated by Tab characters. They are the values of the variable at each simulation step,
- each row of data, associated to a given variable, ends in a carriage return.

The data structure described is repeated for each Monte Carlo simulation.
